# Atypical Functional Connectivity During Unfamiliar Music Listening in Children With Autism

**DOI:** 10.3389/fnins.2022.829415

**Published:** 2022-04-19

**Authors:** Carina Freitas, Benjamin A. E. Hunt, Simeon M. Wong, Leanne Ristic, Susan Fragiadakis, Stephanie Chow, Alana Iaboni, Jessica Brian, Latha Soorya, Joyce L. Chen, Russell Schachar, Benjamin T. Dunkley, Margot J. Taylor, Jason P. Lerch, Evdokia Anagnostou

**Affiliations:** ^1^Institute of Medical Science, Faculty of Medicine, University of Toronto, Toronto, ON, Canada; ^2^Bloorview Research Institute, Holland Bloorview Kids Rehabilitation Hospital, Toronto, ON, Canada; ^3^Department of Diagnostic Imaging, Hospital for Sick Children, Toronto, ON, Canada; ^4^Neuroscience and Mental Health Program, Hospital for Sick Children Research Institute, Toronto, ON, Canada; ^5^Department of Paediatrics, University of Toronto, Toronto, ON, Canada; ^6^Department of Psychiatry, Rush University Medical Center, Chicago, IL, United States; ^7^Faculty of Kinesiology and Physical Education and Rehabilitation Sciences Institute, University of Toronto, Toronto, ON, Canada; ^8^Department of Psychiatry Research, Hospital for Sick Children, Toronto, ON, Canada; ^9^Departments of Psychology and Medical Imaging, University of Toronto, Toronto, ON, Canada; ^10^Department of Medical Biophysics, University of Toronto, Toronto, ON, Canada; ^11^Wellcome Centre for Integrative Neuroimaging, University of Oxford, Oxford, United Kingdom

**Keywords:** autism spectrum disorder, music, familiarity processing, magnetoencephalography, neural oscillation, functional connectivity

## Abstract

**Background:**

Atypical processing of unfamiliar, but less so familiar, stimuli has been described in Autism Spectrum Disorder (ASD), in particular in relation to face processing. We examined the construct of familiarity in ASD using familiar and unfamiliar songs, to investigate the link between familiarity and autism symptoms, such as repetitive behavior.

**Methods:**

Forty-eight children, 24 with ASD (21 males, mean age = 9.96 years ± 1.54) and 24 typically developing (TD) controls (21 males, mean age = 10.17 ± 1.90) completed a music familiarity task using individually identified familiar compared to unfamiliar songs, while magnetoencephalography (MEG) was recorded. Each song was presented for 30 s. We used both amplitude envelope correlation (AEC) and the weighted phase lag index (wPLI) to assess functional connectivity between specific regions of interest (ROI) and non-ROI parcels, as well as at the whole brain level, to understand what is preserved and what is impaired in familiar music listening in this population.

**Results:**

Increased wPLI synchronization for familiar vs. unfamiliar music was found for typically developing children in the gamma frequency. There were no significant differences within the ASD group for this comparison. During the processing of unfamiliar music, we demonstrated left lateralized increased theta and beta band connectivity in children with ASD compared to controls. An interaction effect found greater alpha band connectivity in the TD group compared to ASD to unfamiliar music only, anchored in the left insula.

**Conclusion:**

Our results revealed atypical processing of unfamiliar songs in children with ASD, consistent with previous studies in other modalities reporting that processing novelty is a challenge for ASD. Relatively typical processing of familiar stimuli may represent a strength and may be of interest to strength-based intervention planning.

## Introduction

Autism spectrum disorder (ASD) is a neurodevelopmental disorder characterized by persistent difficulties in social interaction and social communication, and restricted, repetitive patterns of behaviors, interests and activities. In the first description of autism, Kanner described key features present in all of his 11 original cases of children; these were extreme aloneness, lack of interaction, inability to form close emotional ties with others and “an anxious desire for the maintenance of sameness.” This need for sameness often presents as rigorous adherence to particular routines or rituals and resistance to change in the surroundings (such as placement of toys). This subgroup of restricted and repetitive behaviors (RRBs) is currently referred as insistence on sameness (IS) (Szatmari et al., 2006; Bishop et al., 2013).

Both social communication and repetitive behavior domains have been linked to novelty processing. Munson et al. (2008) demonstrated that memory and preference for novelty were predictive of social and communication growth in pre-schoolers with ASD between 4 and 6 1/2 years of age. In contrast, the desire for sameness and resistance to change may highlight a need for preservation and adherence to familiar environments and routines (Szatmari et al., 2006) and a heightened sensitivity to novel stimuli may underlie preference for sameness (Baron-Cohen and Belmonte, 2005).

The effect of familiarity has been investigated in the ASD population in only a few experimental tasks. Most of these studies were on face processing, as understanding socio-emotional difficulties is relevant in this disorder. Faces are salient stimuli critical for social interaction and communication (Jack and Schyns, 2015). Atypical face recognition processing in the ASD population has been reported in different neuroimaging modalities, including event-related potentials (ERPs) (Dawson et al., 2002; Webb et al., 2006, 2010; Batty et al., 2011), magnetoencephalography (MEG) (Leung et al., 2014, 2018; Safar et al., 2018) and fMRI (Pierce et al., 2004; Grelotti et al., 2005; Pierce and Redcay, 2008). Compared to controls, ASD individuals show face recognition impairments in the processing of unfamiliar but not familiar faces (Simmons et al., 2009), although some authors suggest delayed development of the processing of familiar faces (Batty et al., 2011; Webb et al., 2011). Nevertheless, unfamiliar faces have been the preferred stimuli to investigate the altered emotional face processing in ASD. Familiarity has also been related to the need for sameness and repetitive behaviors in ASD (Gomot et al., 2008; Chen et al., 2009; Boyd et al., 2010), suggesting it has a valuable role in helping understand these classic symptoms.

Music is invaluable to the study of human cognition, emotion and underlying brain networks, including familiarity (Koelsch, 2005a,b). Unlike faces, music is an auditory stimulus that interests and motivates many children with ASD (Kanner, 1943). Despite a 50 years history of music therapy in autism (Reschke-Hernández, 2011) and many anecdotal reports of its importance to ASD children (Sacks, 2008), there has been only a handful of fMRI studies on songs or music processing in ASD. These studies examined either emotional processing of music in adults with ASD (Caria et al., 2011; Gebauer et al., 2014) or compared music and language processing in autistic children (Lai et al., 2012; Sharda et al., 2015), given that both music and language share perceptual and neural mechanisms (Fedorenko et al., 2009; Schön et al., 2010; Rogalsky et al., 2011). These studies used different neuroimaging methods, such as fMRI, diffusion tensor imaging (DTI) or multimodal approaches, with a passive listening paradigm, but the familiarity effect was not investigated.

Even though familiar and unfamiliar songs were used separately in the above reports, to our knowledge, there is no neuroimaging study that compared familiar and unfamiliar music listening in individuals with ASD. Understanding the neural mechanisms underlying music familiarity processing is important to understanding the neurobiological substrates of familiarity processing in ASD, but also to inform therapeutics, especially given the potential impact of the construct of familiarity on both social communication and insistence on sameness.

Neuroimaging studies focusing on the processing of familiarity in music listening have been completed in healthy adults (Plailly et al., 2007; Watanabe et al., 2008; Janata, 2009; Pereira et al., 2011; Sikka et al., 2015), in adults with Alzheimer’s disease (Yang et al., 2015; King et al., 2018) and Down syndrome (Virji-Babul et al., 2013). In a recent neuroimaging meta-analysis on familiarity in music listening in healthy adults (Freitas et al., 2018), we found that listening to familiar music demonstrated the highest likelihood of activation in left motor and premotor areas, suggesting an audio-motor synchronization to familiar tunes, anticipating music elements (melody, rhythm harmonic progression, lyrics) in one’s mind. On the other hand, unfamiliar music activated the left insula and the right anterior cingulate cortex, important structures for evaluative judgments (Brattico and Pearce, 2013), potentially deciding whether a song is familiar or novel. Surprisingly, the brain regions related to emotion and reward were not amongst the most consistently activated while listening to familiar songs, as we had expected.

Thus, familiarity and novelty processing relate to core ASD symptoms (social communication deficits and repetitive behaviors) and understanding deficits and strengths in familiarity processing may provide insights into the nature of ASD. Understanding what is impaired and what is preserved in familiarity processing across different sensory modalities will provide important guidance for the development of therapeutic strategies in this population.

With the present research we fill this existing literature gap on music familiarity in ASD and address the following questions: (i) What patterns of functional connectivity are associated with familiarity music processing in typically developing children? We define familiarity as the “feeling of knowing a song,” as described in the literature (Groussard et al., 2010). (ii) Do children with autism differ from typically developing children when neurally processing the songs during familiar or unfamiliar music? (iii) Will atypical network connectivity relate to the insistence on sameness or social communication?

The MEG analyses of this study were guided by the results of the our previous systematic and fMRI neuroimaging meta-analysis (Freitas et al., 2018) which identified the most consistently active brain areas (nodes) when listening to familiar and unfamiliar songs. These areas included the left superior frontal gyrus, SMA, left ventral lateral nucleus of the thalamus, left insula, right cingulate, among others. We selected the top 8 nodes in each condition (familiar and unfamiliar) as our regions of interest (ROI) for connectivity analyses. We chose to analyze two distinct connectivity approaches to MEG data, as one, AEC, reflects coupled fluctuations of signal envelope in neural oscillations and the other, wPLI, measures quick, transient activity related to rapid processes in the brain. These two complementary approaches provide information on different aspects of the dynamics of ongoing neural activity (for a review, please see Engel et al., 2013).

## Materials and Methods

### Participants

Forty-eight youth were included in the study. Twenty-four children and adolescents with ASD (21 males; range: 7 to 14 years, mean = 9.96 years ± 1.54, 20 right handed) sex-matched with 24 typically developing controls (21 males; range 7 to 14 years old, mean = 10.17 ± 1.90, 20 right handed) were recruited from the Province of Ontario Neurodevelopmental Disorders (POND) Network dataset. Exclusion criteria included a history of brain injury and major psychiatric illness for children with ASD, and comorbid psychiatry disorder and first-degree family history of neurodevelopmental disorder for the control group. In addition, for both groups uncorrected vision, blindness, deafness, IQ < 70 and ferromagnetic dental work or metallic implants were also exclusion factors. The study was approved by The Holland Bloorview Kids Rehabilitation Hospital and The Hospital for Sick Children Research Ethics Boards. All participants and their parents gave informed written assent and consent.

### Clinical Evaluation

Participants with ASD had been diagnosed by a registered medical professional and clinical psychologists according to the DSM-5 criteria. Diagnoses were confirmed by the Autism Diagnostic Observation Schedule (ADOS-General or ADOS-2) and the Autism Diagnostic Interview- Revised (ADI-R) (Lord et al., 1994). All participants underwent cognitive testing using the Wechsler Abbreviated Scale of Intelligence—Second Edition (WASI-II) (Wechsler, 2002). Parents were interviewed and completed the Social Communication Questionnaire (SCQ) (Rutter et al., 2003) and the Repetitive Behavior Scale Revised (RBS-R) (Lam and Aman, 2007). Participants were also asked about their musical training (if they had formal musical education). Baseline demographic information and performance on cognitive/behavioral measures are presented in Table 1.

**TABLE 1:** Demographic, diagnostic and cognitive-behavioral assessment measures for ASD and control participants.

	ASD Mean ± SD (*n*)	Controls Mean ± SD (*n*)	Test statistic
			
Total N	24 (3 female)	24 (3 female)	
Male: female	21:3	21:3	
Age	10.0 ± 1.5 (24)	10.17 ± 1.90 (24)	N.S
ADOS2	6.8 ± 2.1 (20)	NA	
WASI-II	98.0 ± 15. (24)	109.0 ± 11.1 (24)	*t* = −2.72, df = 46, *p* = 0.016
SCQ-L	19.3 ± 6.3 (21)	4.2 ± 2.9 (21)	*t* = 9.84, df = 40, *p* = 0.003
RBS-R	14.2 ± 11.6 (24)	1.9 ± 3.7 (24)	*t* = 4.92, df = 46, *p* < 0.001
Music training	8 (24)	6 (24)	N. S
Yes (total N)			
Pitch Discrimination	See Supplementary Figure 1		

*ADOS-2, Autism Diagnostic Observation Schedule- Second Edition; WASI, Wechsler Abbreviated Scale if Intelligence; SCQ, Social Communication Questionnaire- Lifetime; RBS-R, Repetitive Behavior Scale Revised—subscale (Ritualistic/Sameness Behavior).*

### Study Design

There were two visits for this study. In the first visit, participants were asked to bring a set of 10 familiar liked and 10 disliked songs, rating their likability using a Likert scale—ranging from 1 (least liked or most disliked) to 5 (most liked). They also completed a music questionnaire (assessing music preferences, years of training and type instrument played) (Bhatara et al., 2013) and a pitch discrimination test (Stanutz et al., 2014) (see Supplementary Figure 1). The complete list of all familiar songs used in this study is in Supplementary Table 1. During the second visit they completed a hearing screening and the MEG task.

### Music Stimuli

Our music task paradigm was adapted from an fMRI study on music familiarity in healthy adults described by Pereira et al. (2011). Using songs that are listened to and known by the participants, instead of tones, increases ecological value of the experiment. After the first study visit, we selected 8 familiar most-liked songs and 8 familiar most-disliked songs for each participant from their self-selected music list. This selection was based on participants’ likability ratings: songs rated in the extreme positions of the rating scale. Then, we selected the first 30 s of each song, using Audacity 2.1.0 music software program for editing these music excerpts, due to its relevance on listeners’ attention (Brandon Miler, 2016; Crane, 2017; Léveillé Gauvin, 2018). Subsequently, we extracted three musical features (tempo, mode and dissonance) from the 30 s excerpts, using the Matlab Toolbox for Music Information Retrieval (MIR Toolbox) (Lartillot et al., 2008) version 1.6, running on Matlab 2017b (The MathWorks Inc., Natick, MA, United States). This program provided us with an objective measure of the musical features. Two other musical features, genre and presence or absence of lyrics, were classified by auditory inspection. We matched the familiar songs with unfamiliar ones on the following musical characteristics: tempo [(slow (40 to 72 bpm); moderate (72 to 120 bpm) and fast (120 to 208 bpm)]; mode (minor or major); genre (i.e., classic, pop, rock) and presence or absence of lyrics (vocal or instrumental). For all songs with lyrics (503/551 songs), we matched the language of the lyrics. In total we had songs in 5 languages (English, French, Spanish, Portuguese and Hebrew) which were matched for this variable with unfamiliar songs from our data base. The unfamiliar songs were selected from a database of European music, mostly from the Eurovision Song Contest. European music was chosen as, like most North American popular music, it shares the rules and regularities of Western tonal music but is less likely to be familiar to North American participants. In total, for each participant 16 extracts (30 s each) of familiar songs (liked and disliked) were matched with 24 unfamiliar songs. The list of all unfamiliar songs is available in the Supplementary Table 2.

### Choice of Magnetoencephalography Paradigm

The stimulus duration was 30 s consistent with other studies (Janata, 2009; Demorest et al., 2010; Pereira et al., 2011); this choice was related to the uniqueness of our question. Listening to music excerpts and recognizing if that excerpt is familiar or not is a cognitive task that requires time, especially in children. Recognition is a process that develops gradually while the melody unfolds over time (Dalla Bella et al., 2003).

#### Magnetoencephalography Task

**FIGURE 1:**
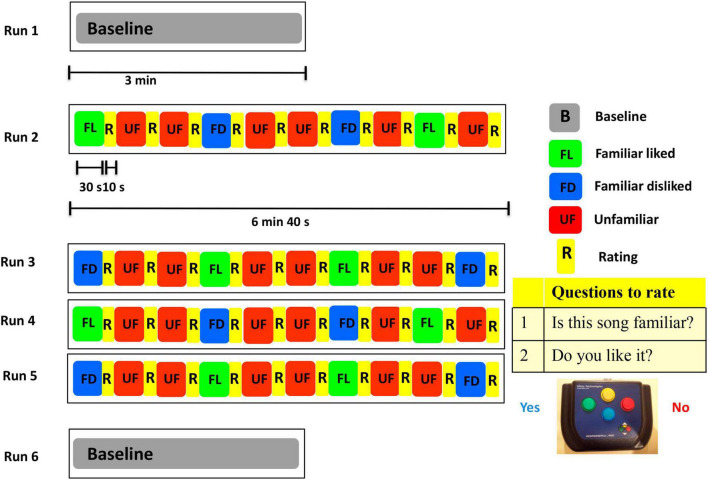
The MEG experimental paradigm.

During the MEG task, stimuli were delivered using Presentation software (Version 18.1, Neurobehavioral Systems, Berkeley, CA, United States). A unique set of 40 song extracts of 30 s each was prepared for each participant. Before entering the MEG scanner, each participant was trained to complete the task with familiar and unfamiliar songs not used in the task experiment. Inside the magnetically shielded room, participants were positioned supine and instructed to maintain visual fixation on an X within a circle projected on the screen, situated ∼70 cm from the participant’s eyes. The whole task consisted of 6 runs: two resting state scans of 3 min each (before and after the music task) and four runs of the music task which included 10 music excerpts in each run (Figure 1). The songs presented were of three different conditions: familiar liked (FL), familiar disliked (FD), and unfamiliar (UF). After hearing each song extract for 30 s through MEG compatible earphones, participants responded to two questions, by pressing left (Yes) or right (No) buttons. The questions were “Do you know this song?” and “Do you like this music?” Participants were instructed to wait until the end of the 30 s before answering, to avoid MEG contamination by the motor response. Ratings took approximately 10 s each. The familiarity rating done during the scanning session was used in the analysis. This task produced a total of 40 trials for each participant.

### Neuroimaging Data Acquisition

The MEG data were obtained using a 151-channel whole-head system with axial gradiometers (CTF MEG International Services LP Coquitlam, Canada). Data were recorded at a 600 Hz sampling rate with a band-pass filter of 1–150 Hz. Localization coils (fiducial markers) positioned at the nasion and left and right pre-auricular points were used to monitor the head position continuously during the task. Trials with greater than 10 mm of head motion were not included (Pang, 2011). The mean head displacement during the task, for the 45 min recording, did not differ between groups (ASD mean = 5.9 mm ± 2.6; TD mean = 4.32 mm ± 1.98; *p* = 0.54). There were also no group differences in motion during the two resting state recordings (ASD mean = 2.6 mm ± 1.3; TD mean = 1.7 mm ± 1.2; *p* = 0.964 and ASD mean = 3.0 mm ± 1.4; TD mean = 2.6 mm ± 1.7; *p* = 0.164). After the MEG session, for co-registration purposes, fiducial coils were replaced by MRI radio-opaque markers. Structural brain MRIs were obtained in all children. In five children a whole brain T1-weighted MRI sequence in a 3.0 T MR scanner (MAGNETOM Tim Trio, Siemens AG, Erlanger, Germany) with a 12-channel head coil was completed. Their brain images were obtained using a high-resolution 3D SAG MPRAGE sequence (PAT, GRAPPA = 2, TR/TE/FA = 2,300/2.96 ms/90, FOV = 28.8 × 19.2 cm, 256 × 256 matrix, 192 slices, slice thickness = 1.0 mm isotropic voxels). The remaining 43 participants were scanned using a 3.0 T MR (PrismaFIT, Siemens Healthineers, Germany) with a head and neck 20-channel coil. Images were obtained using a similar protocol but with 0.8 mm isotropic voxels.

### Neuroimaging Data Preprocessing

The 30 s music trials were epoched into chunks of 10 s (10 + 10 + 10) to avoid rejection of the whole trial due to head movement. This epoching convention was preserved for all analyses. The data epochs for familiar (FL and FD) and unfamiliar (UL and UD) music conditions were selected according to participant’s responses during the MEG task. As a result of variability in each participant’s likeability ratings and the higher number of unfamiliar stimuli in the MEG paradigm, the final number of trials in each music condition differed. The average number of trials for each condition in the ASD group was FL:10.1; FD:5.4; UL:8.9; UD: 15,5; and in the TD group this average was: FL: 9.1; FD: 6.1; UL: 6.2. and UD: 18.4; these numbers of trials did not differ between groups (all *p*s > 0.10). To be considered for data analysis, participants had to have a minimum of 3 trials in each condition (> 90s of data per participant and condition). As the numbers were variable between liked and disliked trials, these two categories were collapsed into familiar and unfamiliar conditions. There were no significant differences in the number of trials included for familiar vs. unfamiliar trials between the two groups. For the TD group, 33.1 ± 8.7 trials were included in analysis, while for the ASD 28.8 ± 9.7 trials were included; there was no significant difference between groups in number of trials in analyses (*t* = −1.5, *p* = 0.12). Independent Component Analysis (ICA) was used to identify the components that reflected ocular artifacts, generated by eye movement and non-ocular artifacts such as cardiac and muscle activity on a trial-by-trial basis for each participant and condition. A maximum of 60 components per participant were visually inspected and artifacts were removed (Muthukumaraswamy, 2013). In the TD group an average of 3.3 ± 0.9 components were dropped, while in the ASD group the average was 3.5 ± 1.0 components dropped. There was no significant difference between groups in number of components dropped (*t* = 1.38, *p* = 0.17).

### Atlas-Guided Source Reconstruction

For MEG data processing we used the FieldTrip software toolbox (Oostenveld et al., 2011) implemented in MATLAB R2017b (The MathWorks Inc., Natick, MA, United States). A single shell head model based on initial fiducial positions using each individual’s MRI scan was constructed and normalized into standard MNI space (ICBM 152; (Fonov et al., 2009, 2011). A total of 92 source (seed) locations were then selected for time-series to be extracted and analyzed. We used the coordinates of 90 sources representing the center of mass of all cortical and subcortical parcels in the Automated Anatomical Labeling (AAL) atlas (Tzourio-Mazoyer et al., 2002) as well as 2 additional coordinates for the accumbens nuclei defined in the Yale BioImage Suite Package^1^. All 92 seeds were projected from standard space into subject space. A linearly constrained minimum variance (LCMV) beamformer (Van Veen et al., 1997) was used to reconstruct broadband time series (“virtual sensors”) for each source location and trial for each subject representing the activity of each of the 92 sources. Beamformers are a type of spatial filter used to estimate activity at a given brain location while suppressing activity from other locations (Van Veen et al., 1997). Our contrasts were binaurally presented and did not detect correlated auditory sources. This could have been due to large analysis windows used with a fairly varied auditory stimuli. Beamformer leakage was corrected using the MEG-ROI-nets toolbox^2^ using the closest method (Colclough et al., 2015) prior to computing connectivity analysis using amplitude envelope correlation. No leakage correction was applied prior to estimation of wPLI, as wPLI is resistant to signal leakage (Vinck et al., 2011). The beamformer projections were computed at the centroid of each AAL region, rather than on a grid.

The broadband time series were filtered using a two-pass FIR filter into five frequency bands for further analysis: theta (4–7 Hz), alpha (8–14 Hz), beta (15–29 Hz), and gamma (30–80 Hz). The gamma frequency was split into gamma 1 (30–55 Hz) and gamma 2 (65–80 Hz). A notch filter was applied at 60 Hz to remove power-line interference (Chimeno and Pallàs-Areny, 2000).

### Assessing Functional Connectivity

We used both amplitude envelope correlation (AEC; Cohen, 2014) and the weighted phase lag index (wPLI; Vinck et al., 2011) to assess connectivity in the music familiarity task amongst *a priori* defined 13 regions of interest (ROI) (from Freitas et al., 2018) and at the whole brain level (AAL—92 regions). The wPLI estimates the degree of phase synchronization based on the magnitude of the imaginary component of the cross- spectrum (Vinck et al., 2011; Lau et al., 2012) and measures quick, transient brain activity (frequency is between 1 to 150 Hz). The amplitude envelope reflects fluctuations in the envelope of spontaneous neural oscillations (Cohen, 2014), and AEC is the correlation over time between seed regions. The AEC has a low frequency (below 0.1 Hz) (but still indexes activity within frequency bands that are invisible to fMRI) and correlates with fMRI activity. These two measures offer complementary information about neural interactions and functional coupling across distinct areas of the brain (Engel et al., 2013). The seed selection was based on our previous meta-analysis on the neural correlates of familiarity in music listening (Freitas et al., 2018; see Supplementary Tables 3, 4). For both ROI and whole-brain analyses, the Hilbert transform was used to obtain time series of the instantaneous phase and amplitude envelope for each source, frequency band and condition. Both AEC and wPLI were calculated within trials.

For the specified ROI analysis, both amplitude and phase-based metrics were used to assess connectivity between thirteen AAL nodes and each of the other 92 AAL nodes, representing the rest of the brain. A 92 × 13 adjacency matrix was created for each trial, frequency band, condition and participant. Following seed analysis, we performed a whole brain analysis, which generated a 92 × 92 adjacency matrix. In both cases, after calculating adjacency matrices, similar subsequent analyses were performed to produce baselined estimates of functional connectivity revealing task-dependent connectomic effects.

For each frequency, condition (familiar, unfamiliar) and subject, functional connectivity matrices were averaged over the music trials. This trial-average was baselined by subtracting the average of 30 s epochs of resting state recording: generating a single functional connectivity matrix per condition, frequency and subject for wPLI and AEC.

### Statistical Analyses of Networks Dynamics

Non-parametric Network-Based Statistics (NBS) (Zalesky et al., 2010) were used for statistical comparison of amplitude and phase connectivity differences within and between-groups while controlling for family wise error rate (FWER) (Zalesky et al., 2010, 2012). This method performs multiple univariate tests (*t*-tests) on all 92 edges (each element of the adjacency matrix) or 92 × 13 for the ROI seed analysis. This yields a *t*-value for each connection in the matrix, the t-values are then thresholded by a primary component-forming threshold and those that exceeded this cut-off were identified and subjected to permutation tests at the network level (5000 permutation in the present study). We set the primary component forming threshold for between and within group comparisons to *t* = 2.75, 3.0, 3.5 for AEC metric and *t* = 3.0 for wPLI measures. These values of the thresholds (around 3.0–3.5) were selected as they are robust levels for significance and hence, commonly reported in the literature. Also, they represent significant, relatively strict, thresholds: *t* = 2.75 corresponds to ∼*p* < 0.005; *t* = 3.00 corresponds to ∼*p* < 0.001. Furthermore, the networks were stable across these thresholds. Statistical significance was assigned at the level of the connectivity component as a whole, defining clusters of functionally integrated nodes that significantly differed between groups or conditions. Statistical correction controlling for false positives due to multiple comparisons was performed within each frequency band for the two types of analysis using Bonferroni correction (*p* < 0.0125 and *p* < 0.025 for between and within groups’ differences, respectively). We corrected only within metric, as the metrics are distinct and look at two quite different aspects of the signal (i.e., amplitude envelopes versus phase). The results obtained using NBS were plotted using the Brain Net Viewer toolbox (Xia et al., 2013).

### Brain-Behavioral Analyses

We performed a correlation analysis across ASD and TD groups between mean network strength (edge weights summed for each individual subject) of significant group difference networks and the scores of the Social Communication Questionnaire (SCQ) scores (Rutter et al., 2003), as well as the ritualistic/sameness subscale of the Repetitive Behavior Scale Revised (RBS-R) (Lam and Aman, 2007) using SPSS 25.0 software (IBM Corp. Released, 2017. IBM SPSS Statistics for Windows, Version 25.0. Armonk, NY: IBM Corp) to explore whether differences seen on connectivity during familiar/unfamiliar music listening were related to core symptom domains of ASD.

## Results

Behavioral results (Supplementary Table 1 and Supplementary Figure 1).

### Magnetoencephalography Results

#### Connectivity Within Groups: Familiar vs. Unfamiliar

##### Regions of Interest Analyses

**FIGURE 2:**
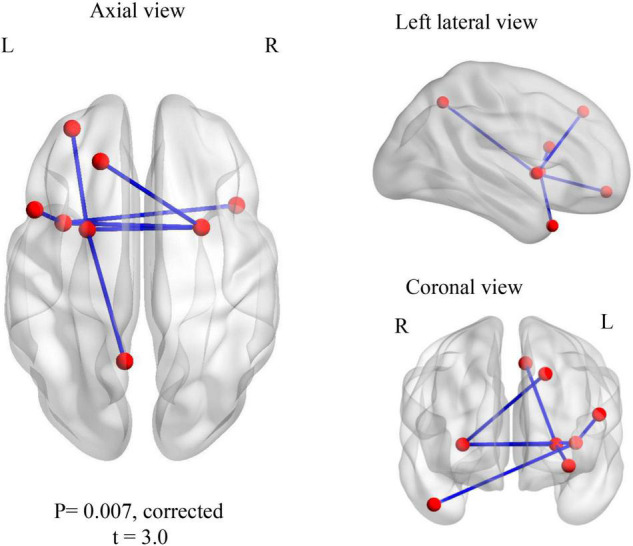
Within TD group contrast: familiar > unfamiliar music. Increased gamma 1 band (30–55 Hz) wPLI phase synchronization during processing of familiar music in TD children.

For the within-group analysis, we compared familiar to unfamiliar music for both ASD and control groups, separately, in both amplitude and phase measures. Increased wPLI synchronization was noted for control children in the gamma 1 frequency band for familiar music > unfamiliar music. The network comprised seven edges and eight nodes (*p*_*corr*_ = 0.007), involving connections in the left hemisphere, the superior and inferior opercular frontal gyri, the putamen, the middle orbital gyrus, the insula and the precuneus, and also the right middle temporal pole and the right putamen (Figure 2). There were no significant differences within the ASD group. For the opposite contrast (unfamiliar > familiar music) there were no significant differences in connectivity in either group.

##### Whole-Brain Analyses

There were no significant differences at the whole brain level in either amplitude or wPLI metrics for any of the 5 frequencies bands tested for either ASD or control group, for the same contrasts.

Regions of Interest and whole brain connectivity within groups comparing the active window with the baseline for ASD and control groups, separately, for familiar and unfamiliar music conditions are reported in Supplementary Tables 5–8.

#### Connectivity Between Groups

##### Regions of Interest Analyses

**FIGURE 3:**
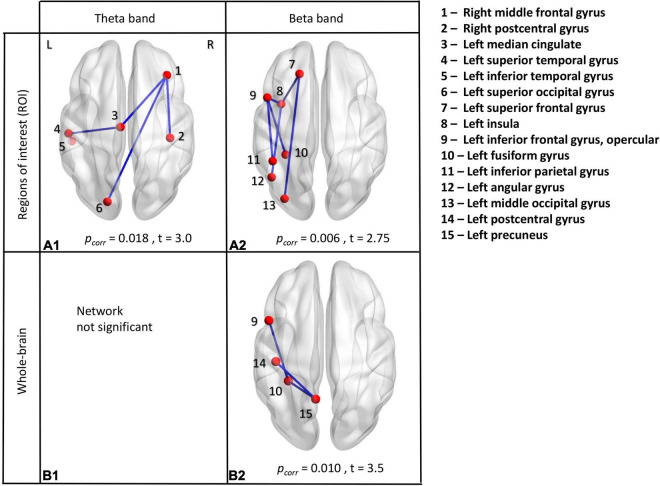
Between-group analyses. ROI analyses in theta frequency band **(A1)** and beta frequency band **(A2)**. Whole-brain analyses in theta frequency band **(B1)** and beta frequency band **(B2)**. All significant results indicated increased connectivity in the ASD group compared to TD during the presentation of unfamiliar music, measured using amplitude envelope connectivity.

We also conducted between-group contrasts for each frequency band and music condition (i.e., familiar and unfamiliar). No main effects for group or condition were found. A difference emerged for the processing of unfamiliar music, where children with ASD showed increased amplitude connectivity in the theta and beta frequency bands compared to controls (*p*_*corr*_ = 0.018 and *p_*corr*_* = 0.0064, respectively). Increased theta connectivity involved four edges and six connected nodes: the right middle frontal and right post-central gyri, the left superior occipital gyrus, the left mid-cingulum and the left superior and inferior temporal gyri (Figure 3A1). Increased beta connectivity involved six edges and seven connected nodes, all left lateralized: the superior frontal gyrus, the opercular frontal, the insula, the fusiform gyrus, the inferior parietal gyrus, the angular gyrus and the middle occipital gyrus (Figure 3A2). No significant differences emerged for the between-group contrasts in the familiar music condition. There were no significant differences in other frequencies bands.

##### Whole Brain Analyses

We also performed between-group contrasts for each frequency band and music condition (i.e., familiar and unfamiliar) using a whole-brain analysis. No significant group or condition main effects were seen. Again, a difference emerged only for the processing of unfamiliar music. Not in theta frequency band (Figure 3B1), as in ROI analysis, but in beta frequency band. ASD children showed increased amplitude connectivity in the beta frequency band compared to controls (*p*_*corr*_ = 0.010). Increased beta connectivity involved three edges and four connected nodes, all in the left hemisphere: the opercular part of the inferior frontal gyrus, the postcentral gyrus, the fusiform gyrus and the precuneus (Figure 3B2). No other significant differences emerged for the between-group contrasts in other frequencies bands.

#### Interaction Effects in Regions of Interest Analysis

**FIGURE 4:**
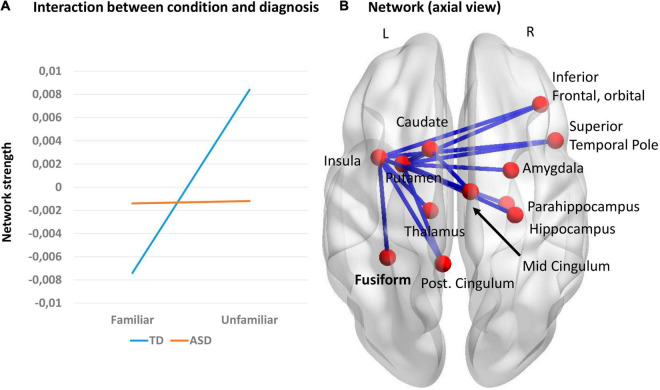
**(A)** Interaction effects between music condition and group; **(B)** Network representing the significant interaction in the alpha frequency band, using the wPLI metric (*p* = 0.023).

We also explored interaction effects using a 2 (group: ASD, TD) × 2 (music condition: familiar, unfamiliar) mixed design ANOVA using NBS, in all five frequency bands, using both amplitude and wPLI metrics. The primary threshold was set to *F* = 7. Significant results were found using the wPLI metric in the alpha frequency band. The mean network connectivity was different for ASD and TD groups depending on music familiarity (*p* = 0.023). The TD group had greater connectivity in the unfamiliar condition. No effect was seen in the ASD group (Figure 4A). A significant interaction for TD children is represented by a network consisting of 15 edges and 12 nodes (Figure 4B). This network was anchored in the left insula and putamen, but with nodes in both hemispheres. No significant results were found using the amplitude metric.

#### Brain-Behavior Relations

We had three statistically significant networks emerge from between group differences, all in the unfamiliar music condition (network 1 = ROI _theta, network 2 = ROI_ beta, and network 3 = WB _theta). We selected the global strength (average of the strengths of all nodes) of these networks as one of the most fundamental measures of brain topology assessed by graph theory (Mijalkov et al., 2017). First, we correlated these network connectivity strengths with the ADOS calibrated severity score within the ASD group, but did not find any significant correlations (network 1: *r* = 0.008, *p* = 0.975; network 2: *r* = 0.334, *p* = 0.191; and network 3: *r* = 0.147, *p* = 0.574; uncorrected). We also explored if RBS-R-subscale IV (sameness) scores in ASD participants were correlated with network connectivity strength, but no significant results were found in any of the three networks (network 1: *r* = −0.037, *p* = 0.889; network 2: *r* = 0.031, *p* = 0.906 and network 3: *r* = −0.307, *p* = 0.230). Lastly, no significant correlations were noted between SCQ and RBS and network connectivity strength within either group (Supplementary Table 9).

## Discussion

In this study, we examined the neural correlates of the processing of familiarity in music listening in typically developing and autistic children using MEG, at the macro-scale level to fill an existing literature gap. For this, we performed connectivity analyses within and between groups using wPLI and AEC measures, at both ROI and whole-brain level. In the following sections we will discuss our findings with respect to these two groups.

### Typically Developing Children

We demonstrated increased gamma wPLI synchronization in typically developing children for familiar music > unfamiliar music. Listening to familiar music increased phase connectivity in a network that consisted of frontal, parietal, temporal and subcortical areas. The function of these regions has been associated with expressive language (the left inferior opercular frontal gyrus), memory (left superior frontal gyrus), emotional processing (right temporal pole and left insula) (Olson et al., 2007), mental imagery, memory recollection, information integration and visuo-spatial imagery (precuneus) (Cavanna and Trimble, 2006). The motor areas activated here (i.e., the left and right putamen and left caudate) can reflect auditory-motor synchronization to music elements (Chen et al., 2008; Freitas et al., 2018). More specifically, Grahn (2009) demonstrated that the basal ganglia are crucial for rhythm processing, with a role linked to internal generation of the beat. It was not surprising that auditory areas were not included in our network, as the sound was delivered bilaterally, and was equated for familiar and unfamiliar stimuli, so in the contrasts no auditory cortex effects would be expected. Our resulting network for control children was very similar to the results of music familiarity processing in the typical adult neuroimaging meta-analysis (Freitas et al., 2018).

Our connectivity findings in the TD group were found in gamma band frequency only. The gamma band oscillations (30–100 Hz) have been shown to be correlated with various cognitive processes (Engel et al., 2001), the most prominent of which is memory (encoding and performance) (Sederberg et al., 2003) and perceptual binding (Rodriguez et al., 1999). As recognizing a familiar song involves linking and integrating memory processes with auditory percepts, our results align with this explanation that gamma synchrony mediates the coupling of functionally specialized regions involved in music listening.

### Children With Autism Spectrum Disorder

In the children with ASD there were no differences in the MEG metrics between the processing of familiar and unfamiliar music. However, between ASD and TD groups our results demonstrated consistent, significant differences in the unfamiliar music condition at both ROI and whole-brain analyses, assessed by amplitude envelope connectivity (AEC). ASD children showed increased connectivity compared to controls, while the processing of familiar music was similar in the two groups.

This is the first neuroimaging study focusing on understanding familiarity in music in ASD; thus, there is no comparative study available. Nevertheless, neuroimaging literature on the processing of familiarity in faces in ASD individuals has shown atypicalities. It has been suggested that there may be impaired processing of unfamiliar faces, with no deficit or delayed development of familiar faces (Simmons et al., 2009). Pierce and Redcay (2008) reported a selective deficit in fusiform function in response to adult stranger faces, but no atypicalities in the fusiform in response to familiar faces. One possible explanation for this finding was either reduced or enhanced attention or motivation to attend to unfamiliar and familiar faces, respectively. Our findings of atypical processing of unfamiliar music in ASD children could be consistent with this interpretation. Our task (listening to songs for 30 s and deciding if familiar or not) required sustained attention. In addition, repetition increases perceptual fluency (Alter and Oppenheimer, 2009; Joye et al., 2016), or the ease of processing stimuli. As such, we could interpret that unfamiliar songs would require more effort to process. As an example, Pernet et al. (2003) demonstrated in the visual recognition domain using ERPs that the more familiar an object is, the fewer cognitive resources are required. Another interpretation is that ASD children showed reduced neural adaptation-like effects (also referred as habituation or repetition suppression) to unfamiliar music. Neural adaptation is defined by decreasing sensitivity to a repeatedly presented stimulus, whereby the sensory system codes for the derivative or change in the stimulus, priming the individual for changes in the environment: for example, no longer being consciously aware of clothes on the skin over time. In ASD neural adaptation is attenuated in sensory domains, at multiple levels in time and space, from short time scales to long, and in multiple sensory pathways, from the level of neurons, through to the macroscopic BOLD signal, with reduced cortical adaptation effects to sensory stimuli (Turi et al., 2015; Noel et al., 2017), including the auditory domain (Millin et al., 2018). In other words, the ASD brain processes novelty differently compared with the typical brain, driven by possibly impaired neural adaptation and deficits in the dynamic range of neural responsiveness.

Consistent with this, 90% of individuals with autism present atypical sensory experiences: These atypicalities affect every sensory modality and also deficits in multisensory integration (Tomchek and Dunn, 2007; Robertson and Baron-Cohen, 2017). This raises the question if the absence of processing differences between familiar and unfamiliar music results from atypical modulation of sensory processing by high-order cognitive mechanisms. In ASD, several learning challenges, including stimulus overselectivity, have been related to the processing of novel stimuli (Kelly et al., 2015). Stimulus overselectivity could be due to either attentional or performance deficits. The theoretical perspective of “attention deficit” posits that there is an attention abnormality (a narrowness of attention or a hyper-attentiveness to selective stimuli) (Dube, 2009). In our study because of exposure to the same category of stimuli, children with ASD could have given more attention to their familiar music. Regarding the performance deficits theory, it attributes performance problems to an “over-sensitive” comparator mechanism (Reed, 2011). This comparator mechanism is over-sensitive to small differences in importance between stimuli and will respond to a narrow set of stimuli. Under this theory, our children with ASD directed their behavior to familiar music at the expense of unfamiliar music.

Another theory suggests that ASD individuals present deficits in Bayesian prediction processes in information processing (Pellicano and Burr, 2012). This Bayesian framework posits that these individuals see the world more accurately because they lack the modulation by prior experience (top-down mechanisms). The “hypo-prior” (attenuated Bayesian priors) may lead to difficulties in using information from the remote past to match with incoming stimuli. In this case, we could speculate that increased (atypical) connectivity during processing of unfamiliar songs, compared to controls, could be due to a lack of priors (i.e., the children with ASD cannot relate the current experiences to previous experience).

Another consideration is whether music training would have affected familiarity processing. Dalla Bella et al. (2003) reported that music training affects familiarity judgments, and musicians recognize familiar songs in fewer notes than non-musicians. However, our participants did not show group differences in music training (Table 1) or pitch discrimination abilities (an ability correlated with music training) (Supplementary Figure 1), meaning that this was not a selection bias that could be a potential confounding factor.

In this study the differences in the unfamiliar condition in ASD compared to controls were in theta and beta frequencies. Theta oscillations are associated with long-range communication between brain areas implicated in various cognitive processes, such as imitation, language acquisition, working memory, attention, cognitive control and emotional arousal (Engel and Fries, 2010; Kikuchi et al., 2015). Consistent with the existing literature, these theta-band dependent alterations were identified across nodes involving long distance connections that comprise large-scale networks, integrating and coordinating information between frontal, parietal and occipital brain regions. Synchrony in beta oscillations has also been found in long-range cortical interactions related to sensorimotor function, primary-motor integration and switching from the “status quo” (Engel and Fries, 2010; Siegel et al., 2012). We found increased beta band synchrony in frontal-temporal, fronto-occipital and parieto-temporal connections with a left lateralization. More specifically, both networks (see Figures 3A2,B2) included part of the Broca’s area, the pars opercularis of the inferior frontal gyrus (IFG; Brodmann area 44). This area is a component of the motor articulatory network involved in speech production, phonological (Snell, 2010) and semantic processing (Roskies et al., 2001). Moreover, the pars opercularis has been found to be one of the human mirror neurons regions. It is active in motor imagery, imitation and action observation of distal hand and mouth actions (Rajmohan and Mohandas, 2007). Listening to songs requires processing verbal (lyrics) and musical (tunes) components, as well as motor preparation to sing along, dance or tap to the beat. One interpretation could be that children with ASD, compared to controls, showed impaired processing of these left-hemisphere brain functions when listening to unknown songs. Many fMRI studies have reported that ASD individuals lack left lateralization in structure and function of brain areas involved in language (Boddaert et al., 2003; Knaus et al., 2010; Nielsen et al., 2014) which can support this interpretation.

Our findings of differences in theta and beta frequencies are of particular interest in the context of music processing. Previous MEG studies in the auditory domain have implicated theta as well as beta-band activity in the detection of pitch changes (Florin et al., 2017). Also, theta oscillations are important for temporal integration and for the detection of sounds (Ng et al., 2012; Florin et al., 2017). One potential explanation could be that children with ASD would have atypical processing of pitch compared to TD children, even though we did not find behavioral differences between groups in pitch discrimination ability. In addition, beta band is implicated in motor functions, and motor atypicalities are frequently reported in individuals with ASD (Williams et al., 2004; Fournier et al., 2010; Buard et al., 2018), implicating the mμ and beta rhythms during a fine motor imitation task in ASD adolescents. Listening to music is a sensorimotor experience in which we recognize a pulse of a rhythm pattern and naturally synchronize to that beat (through foot tapping or clapping). One could hypothesize that ASD children lack typical motor synchronization and entrainment to unfamiliar songs, but this requires further investigation.

The between group differences in theta and beta bands were seen in amplitude envelope connectivity. This measure, which correlates fluctuations in regional neuronal activity, is much slower than wPLI, and networks defined by AEC closely align with resting state networks seen with fMRI (Biswal et al., 1995; Brookes et al., 2011). The slow timescale (<0.1 Hz) of AEC can provide complementary insights of local signal power that regulates the activation of neural populations underlying large scale cortical interactions (Siegel et al., 2012).

When we explored the interaction between familiarity and groups, comparing the effects of connectivity (network strength) in familiar versus unfamiliar music in ASD versus TD children (using wPLI), we saw a pattern of increased connectivity strength in the unfamiliar condition compared to familiar stimuli in TD in the alpha frequency, but no effects in the ASD group. The resulting network is important for processing music familiarity in TD and comprised nodes of the limbic system, anchored in the left insula and putamen, and including the right amygdala, parahippocampus, hippocampus, mid and post cingulum, motor areas (caudate and putamen) and fusiform gyrus. The insulae are important in processing stimuli which are salient, related to the salience network, and atypical insular function has been reported in ASD (Uddin et al., 2013). The fact that the main hub of this network was the left insula suggests that for the TD children, the familiar songs had far more salience for them, capturing their attention, but that this was not the case for the children with ASD. This is also consistent with the work by Odriozola et al. (2016) and Leung et al. (2014), who showed that for faces the right insula activity was atypical in ASD.

It was also interesting to find brain areas related to face processing in this interaction network when processing music familiarity, as recognizing a song also implies recognizing the singer’s voice. There is emerging evidence showing that when we hear a familiar voice, even without seeing the face, our visual face processing brain areas become active (Von Kriegstein et al., 2005; Von Kriegstein and Giraud, 2006; Blank et al., 2011). This cross-modal activation is fast, automatic and well supported by neural circuits, which have been shaped by multisensory stimuli. For example, in an MEG study with typically adults, Schall et al. (2013) investigated early auditory processing of familiar voices and showed that it is facilitated by visual mechanisms.

Alpha wPLI synchronization, as reported in our interaction effect, has been implicated in visual perception in TD individuals (Freunberger et al., 2008; Palva and Palva, 2011) and also plays a key role in cognitive functions, coordinating neuronal processing (Palva and Palva, 2007, 2011). The effect we see here in TD but not in ASD children suggests that individuals with ASD process novel songs differently due to deficits in neural adaptation-like effects.

Lastly, the lack of association between differences in connectivity and measures of core symptom domains was disappointing. This may be partially explained by the fact that processing of familiar stimuli was relatively preserved and as such unlikely to be associated with down-stream behavioral effects. Another consideration is heterogeneity in both the recognition of familiarity, in the ASD symptoms measures and in the ASD population in general.

## Limitations and Future Work

There are some potential limitations of the present study. The first one relates to the sample size. Forty eight participants (24 per group), although aligned with previous MEG studies in autistic children (Gandal et al., 2010; Roberts et al., 2011; Safar et al., 2018; Yuk et al., 2018), is relatively small in the current climate of big data. However, the complexity of the study, with the multiple visits and assessments was a limiting factor. The second limitation is that our clinical and control groups were not matched on IQ (Table 1), which was significantly lower in our clinical group yet still in the average range. This is, however, not atypical in studies with those with ASD, as on average there is an IQ difference. Equating for IQ then makes the ASD group less representative of what is found generally and the results less generalizable.

A final limitation is the exclusion of the cerebellum from our analysis plan due to technical and methodological reasons. The cerebellum is important for sensorimotor, cognitive and emotional processing (Buckner, 2013) and plays a key role in rhythm and timing processes (Schmahmann, 2004; Nozaradan et al., 2017), but MEG signals from the cerebellum are difficult to record and subject to considerable artifact and distortion (Ioannides, 2005; Muthukumaraswamy, 2013). It would also be important for future studies to assess the rhythm abilities of participants as successful recognition of familiar songs builds on melody (pitch) and rhythm identification (Peretz and Coltheart, 2003; Volkova et al., 2014), and differences in these skills may impact the findings.

Our results on relatively preserved processing of familiar songs in ASD cannot address whether this is specific to music networks, as it could be domain-general to all familiar auditory stimuli. Future research could compare the processing of familiar music to the processing of familiar speech or familiar environmental sounds to shed light on this matter.

## Conclusion

This study provides the first evidence of brain connectivity patterns involved in familiarity in music listening in both typically developing and autistic children. Our results revealed atypical processing of unfamiliar songs in children with ASD. During the processing of unfamiliar music, we demonstrated increased theta and beta band task-dependent connectivity in children with ASD compared to controls. The effects of connectivity (network strength) between familiarity and groups showed increased alpha connectivity strength in unfamiliar condition compared to familiar stimuli in TD but no effect was seen in the ASD group. These results, in addition to adding valuable information to the growing literature on atypical brain connectivity in the ASD population, inform future research in the field of learning and on the neurobiological correlates of music familiarity in autism that may guide the development of music-based interventions. Future work is needed to replicate and expand our findings throughout development, both in typical development and children with ASD.

## Data Availability Statement

The raw data supporting the conclusions of this article will be made available by the authors, without undue reservation.

## Ethics Statement

The studies involving human participants were reviewed and approved by the Research Ethics Board of both Holland Bloorview Kids Rehabilitation Hospital and Hospital for Sick Children. Written informed consent to participate in this study was provided by the participants’ legal guardian/next of kin.

## Author Contributions

CF participated in the task design, data recruitment, data collection, data analysis, results interpretation, and manuscript writing. SW participated in the data analysis. BH participated in the data analysis and interpretation of the results. AI, LR, SF, and SC participated in the data recruitment and data collection. JC, RS, BD, MT, JL, and EA participated in the task design, data analysis, results interpretation, and manuscript editing. JB and LS participated in discussion of the results and this manuscript. All authors contributed to the article and approved the submitted version.

## Conflict of Interest

RS is on the Professional Advisory Board of Ironshore Pharmaceutical and CHADD. EA has received consultation fees from Roche and Quadrant; industry funding from Roche, SynapDx and Sanofi-Aventis; in kind industry support from AMO Pharma and CRR, royalties from APPI and Springer International Publishing; editorial honoraria from Wiley. The remaining authors declare that the research was conducted in the absence of any commercial or financial relationships that could be construed as a potential conflict of interest.

## Publisher’s Note

All claims expressed in this article are solely those of the authors and do not necessarily represent those of their affiliated organizations, or those of the publisher, the editors and the reviewers. Any product that may be evaluated in this article, or claim that may be made by its manufacturer, is not guaranteed or endorsed by the publisher.

## Footnotes

1
https://medicine.yale.edu/bioimaging/suite/mni2tal/

2
https://github.com/OHBA-analysis/MEG-ROI-nets
