# Supplementary material for: Atypical Functional Connectivity During Unfamiliar Music Listening in Children With Autism
Source: Front Neurosci. 2022 Apr 19;16:829415. doi: 10.3389/fnins.2022.829415 (PMC9063167; doi:10.3389/fnins.2022.829415)
Supplement: Supplementary file 1 [file Table_1.DOCX]

Supplementary Material

Atypical Functional Connectivity during Unfamiliar Music Listening in Children with Autism

**Carina Freitas^1,2^**^*^**, Benjamin A. E. Hunt^3,4^, Simeon Wong^3,4^, Leanne Ristic^2^, Susan Fragiadakis^2^, Stephanie Chow^2^, Alana Iaboni^2^, Jessica Brian^2,5^, Latha Soorya^6^, Joyce Chen^7^, Russell Schachar^8^, Benjamin Dunkley^3,4^, Margot J. Taylor^1,3,4,9^, Jason P. Lerch^4,10, 11^, Evdokia Anagnostou^1,2,4,5^**

*** Correspondence:** Carina Freitas: [carina.debarrosfreitas@mail.utoronto.ca](mailto:carina.debarrosfreitas@mail.utoronto.ca)

**Supplementary Table 1**: List of familiar songs used to set up MEG paradigm.

|  | **Name of the song** | **Artist** | **Nationality** | **Language lyrics** | **Genre** | **Tempo (group)** | **Tempo**  **(bpm)** | **Mode (V)** | **Mode** |
| --- | --- | --- | --- | --- | --- | --- | --- | --- | --- |
| 1 | (You're) having my baby | Paul Anka, Odia Coates | Canadian | English | Rock | Fast | 152 | 0.20 | Major |
| 2 | 679 | Fetty Way | American | English | Rap | Fast | 129 | -0.13 | Minor |
| 3 | #thatPOWER | will.i.am, Justin Bieber | American | English | EDM | Fast | 128 | 0.15 | Major |
| 4 | 0 to 100 | Drake | Canadian | English | Rap/hip hop | Fast | 180 | -0.11 | Minor |
| 5 | 1-800-273-8255 | Logic, Alessia Cara, Khalid | American | English | Rap/hip hop | Moderate | 105 | 0.06 | Major |
| 6 | 24K magic | Bruno Mars | American | English | Pop/disco/funk | Moderate | 107 | 0.19 | Minor |
| 7 | 50 ways to say goodbye | Train | American | English | Alternative rock | Fast | 140 | 0.03 | Minor |
| 8 | 500 miles | The Proclaimers | Scottish | English | Pop/voice/bass | Fast | 132 | 0.01 | Major |
| 9 | 5th symphony | Beethoven | German | not applicable | Classical | Moderate | 92 | 0.01 | Minor |
| 10 | 7 years | Lukas Graham | Danish | English | Pop | Slow | 65 | 0.05 | Minor |
| 11 | A bicyclette | Bourvil | French | French | Pop | Fast | 139 | 0.16 | Major |
| 12 | A dream is a wish your heart makes (Cinderella movie) | Disney | American | English | Classical | Moderate | 116 | -0.02 | Minor |
| 13 | A sky full of stars | Coldplay | British | English | Pop | Fast | 125 | 0.30 | Major |
| 14 | A whole new world (Aladdin movie) | Disney | American | English | Musical | Slow | 62 | 0.36 | Minor |
| 15 | ABC song | Nursery Rhymes &Kids Songs | American | English | Cartoons | Moderate | 110 | 0.09 | Major |
| 16 | Above all | Michael W. Smith | American | English | Church/pop | Slow | 66 | 0.16 | Major |
| 17 | Achy breaky heart | Billy Ray Cyrus | American | English | Pop country | Fast | 121 | -0.21 | Minor |
| 18 | Act naturally | Beatles | British | English | Rock | Fast | 187 | 0.24 | Major |
| 19 | Afraid | The Neighbourhood | French | English | Hip hop | Fast | 170 | -0.12 | Minor |
| 20 | Africa | Karl Wolf | Canadian | English | Pop | Fast | 126 | 0.36 | Major |
| 21 | Ain't no mountain high enough | Marvin Gaye, Tammi Terrel | American | English | Soul | Fast | 125 | 0.10 | Major |
| 22 | Ain't nobody | Felix Jaehn | German | English | House | Moderate | 119 | 0.14 | Major |
| 23 | All about that bass | Meghan Trainor | American | English | Pop/doo-wop | Moderate | 90 | 0.01 | Major |
| 24 | All I do is win | DJ Kahled | American | English | Hip hop | Moderate | 100 | -0.05 | Minor |
| 25 | All the way | Jacksepticeye | Irish | English | Rap | Fast | 128 | -0.16 | Minor |
| 26 | All time low | Jon Bellion | American | English | Pop rock | Moderate | 93 | 0.08 | Major |
| 27 | All together now | Beatles | British | English | Pop | Moderate | 96 | 0.04 | Major |
| 28 | All you need is love | Beatles | British | English | Pop | Moderate | 97 | 0.34 | Major |
| 29 | Alone | Marshmello | American | English | Electro dance | Moderate | 95 | 0.22 | Major |
| 30 | Alphabet medley | Nursery Rhyme | American | English | Cartoons | Fast | 126 | 0.09 | Major |
| 31 | Amazed | Lonestar | American | English | Pop | Slow | 71 | 0.13 | Major |
| 32 | American beauty | [Drew Holcomb](https://www.youtube.com/channel/UCphGvmrS31gsmNv2p47mzoQ) | American | English | Pop | Moderate | 112 | 0.03 | Minor |
| 33 | Angel dance | Robert Plant | British | English | Rock | Fast | 167 | -0.11 | Major |
| 34 | Animals | Maroon 5 | American | English | Pop | Moderate | 96 | -0.20 | Minor |
| 35 | Animals | Martin Garrix | Dutch | English | Dance | Fast | 128 | -0.29 | Minor |
| 36 | Anything | Hedley | Canadian | English | Pop rock | Moderate | 103 | 0.16 | Major |
| 37 | As long as you love me | Justin Bieber | Canadian | English | Pop | Slow | 70 | 0.00 | Minor |
| 38 | Ave Maria | Charlotte Church | British | English | Classic | Fast | 136 | 0.02 | Minor |
| 39 | Ba ba ba banana | Splash'N Boots | Canadian | English | Children | Fast | 139 | -0.15 | Minor |
| 40 | Baa baa black sheep have you any wool | Nursery Rhyme | American | English | Cartoons | Moderate | 111 | 0.15 | Major |
| 41 | Baby | Justin Bieber, Ludacris | Canadian | English | Pop | Fast | 130 | 0.30 | Major |
| 42 | Baby mine | Disney | American | N.A. | Soundtrack | Slow | 53 | -0.19 | Minor |
| 43 | Baby one more time | Britney Spears | American | English | Pop | Fast | 185 | -0.10 | Minor |
| 44 | Back in black | AC/DC | Australian | English | Hard rock | Moderate | 91 | 0.04 | Major |
| 45 | Backflip | Casey Veggies | American | English | Hip hop | Fast | 195 | -0.01 | Minor |
| 46 | Bad blood | Taylor Swift, Kendrick Lamar | American | English | Pop rap | Fast | 170 | -0.08 | Major |
| 47 | Bad romance | Lady Gaga | American | English | Pop | Moderate | 119 | 0.08 | Major |
| 48 | Bang bang | Jessie J, Ariana Grande, Nicki Minaj | British | English | Funk | Fast | 150 | 0.15 | Major |
| 49 | Batman soundtrack | Prince | American | English | Soundtrack | Fast | 168 | -0.01 | Minor |
| 50 | Bear band serenade | Disney | American | English | Soundtrack | Fast | 132 | 0.24 | Major |
| 51 | Beds are burning | Midnight Oil | Australian | English | Rock | Moderate | 119 | -0.18 | Minor |
| 52 | Between the raindrops | Lifehouse | American | English | Pop | Moderate | 107 | 0.02 | Major |
| 53 | Bingo | Super Simple Songs | Canadian | English | Cartoons | Fast | 161 | 0.31 | Major |
| 54 | Black magic | Little Mix | English | English | Pop | Moderate | 112 | 0.17 | Major |
| 55 | Black SpiderMan | Logic, Damian Lemar Hudson | American | English | Rap/hip hop | Moderate | 93 | 0.02 | Minor |
| 56 | Blackbird | Beatles | British | English | Rock | Moderate | 94 | 0.00 | Minor |
| 57 | Blank | Disfigure | British | N.A. | Electro house | Fast | 140 | 0.02 | Major |
| 58 | Blue (da ba dee) | Eiffel 65 | Italian | English | Electronic | Fast | 126 | 0.04 | Minor |
| 59 | Body language | Queen | British | English | Pop rock | Fast | 129 | 0.23 | Major |
| 60 | Body like a back road | Sam Hunt | American | English | Pop | Slow | 66 | 0.12 | Major |
| 61 | Bones | Ginny Blackmore | New Zealandese | English | Pop | Moderate | 77 | 0.26 | Major |
| 62 | Books are fun | Barney & Friends | American | English | Cartoons | Fast | 137 | 0.29 | Major |
| 63 | Boom boom pow | The Black Eyed Peas | American | English | Electro | Fast | 123 | -0.11 | Minor |
| 64 | Boomerang | Jojo Siwa | American | English | Pop | Moderate | 115 | 0.09 | Major |
| 65 | Booty | Jennifer Lopez | American | English | Pop | Fast | 129 | -0.01 | Minor |
| 66 | Break my mind | DAGames | American | English | Rock | Fast | 130 | 0.11 | Major |
| 67 | Broken wings | Mr Mister | American | English | Pop 80s | Moderate | 103 | -0.08 | Minor |
| 68 | Cache-cache | Maxime Landry | Canadian | English | Pop | Moderate | 116 | 0.20 | Major |
| 69 | Cake | Flo Rida, 99 Percent | American | English | Rap, hip hop | Moderate | 105 | 0.31 | Major |
| 70 | Cake by the ocean | DNCE | American | English | R & B | Moderate | 119 | -0.26 | Minor |
| 71 | California girls | Katy Perry, Snoop Dogg | American | English | Pop | Fast | 125 | -0.16 | Minor |
| 72 | Call me maybe | Carly Rae Jepsen | Canadian | English | Pop | Moderate | 107 | 0.20 | Major |
| 73 | Can it fix it | Bob the builder | British | English | Cartoons | Fast | 140 | 0.22 | Major |
| 74 | Can you feel my heart | Bring Me The Horizon | British | English | Punk Rock | Fast | 170 | -0.23 | Minor |
| 75 | Candyland | Tobu | Latvian | N.A. | Electro house | Moderate | 102 | 0.31 | Major |
| 76 | Can't feel my face | The Weeknd | Canadian | English | Pop | Moderate | 110 | -0.22 | Minor |
| 77 | Can't stop the feeling | Justin Timberlake | American | English | Pop | Moderate | 119 | -0.17 | Minor |
| 78 | Capture of Triiliam | Disney | American | N.A. | Soundtrack | Moderate | 98 | -0.02 | Minor |
| 79 | Carpe diem | Green Day | American | English | Rock | Fast | 140 | -0.16 | Minor |
| 80 | Castle on the hill | Ed Sheeran | British | English | Pop | Fast | 135 | 0.09 | Major |
| 81 | Centuries | Fall Out Boy | American | English | Pop funk | Moderate | 117 | -0.16 | Minor |
| 82 | Chandelier | Sia | Australian | English | Pop | Moderate | 116 | -0.25 | Minor |
| 83 | Cheap thrills | Sia, Sean Paul | Australian | English | Pop | Moderate | 90 | 0.03 | Major |
| 84 | Cheerleader | OMI | Jamaican | English | Pop | Moderate | 78 | -0.02 | Minor |
| 85 | Cherry bomb | The Runaways | American | N.A. | Rock | Fast | 134 | 0.04 | Major |
| 86 | Chop suey! | System of a Down | American | English | Metal alternative | Fast | 130 | -0.05 | Minor |
| 87 | Christ arose (low in the grave he lay) | Robert Lowry | American | English | Church/pop/choir | Moderate | 81 | 0.18 | Major |
| 88 | Chum 2 | Earl Sweatshirt | American | English | Alternative rock | Moderate | 116 | 0.00 | Minor |
| 89 | Cinquante fois | Bruno Pelletier | Canadian | English | Pop | Moderate | 88 | -0.01 | Major |
| 90 | Circle of life (Lion King movie) | Carmen Twillie | American | Zulu | Ethnic | Moderate | 82 | -0.05 | Minor |
| 91 | Closer | The Chainsmokers | American | English | Pop | Moderate | 95 | 0.05 | Major |
| 92 | Cloud 9 | Itro & Tobu | Latvian | N.A. | Electro House | Fast | 128 | 0.20 | Major |
| 93 | Cold | Maroon 5, Future | American | English | Pop | Moderate | 119 | -0.01 | Minor |
| 94 | Cold water | Major Lazer | American | English | Pop | Moderate | 73 | -0.07 | Minor |
| 95 | Come and get your love | Redbone | American | English | Pop | Moderate | 107 | 0.12 | Major |
| 96 | Come on Eileen | The Clash | British | English | New wave | Moderate | 106 | -0.06 | Major |
| 97 | Come with me now | Longos | South African | English | Alternative rock | Moderate | 104 | 0.03 | Major |
| 98 | Company | Justin Bieber | Canadian | English | Pop | Moderate | 82 | 0.00 | Major |
| 99 | Congratulations | Post Malone, Quavo | American | English | Rap/hip hop | Slow | 122 | 0.02 | Minor |
| 100 | Controlla | Alex Aiono | American | English | Pop | Slow | 65 | -0.28 | Minor |
| 101 | Cotton eye Joe | Rednex | Swedish | English | Pop country | Fast | 132 | 0.22 | Major |
| 102 | Counting stars | OneRepublic | American | English | Folk pop | Fast | 122 | 0.19 | Minor |
| 103 | Crown of love | Arcade Fire | Canadian | English | Rock | Slow | 70 | 0.09 | Major |
| 104 | Cruisin' for a bruisin' | from Teen Beach Movie | American | English | Pop | Moderate | 82 | -0.18 | Major |
| 105 | Cucumber | N. A. | Russian | N.A. | Instrumental | Fast | 141 | 0.04 | Major |
| 106 | Dazed and confused | Led Zeppelin | British | English | Pop | Moderate | 114 | -0.14 | Minor |
| 107 | Deep thoughts | Disney | American | English | Soundtrack | Fast | 121 | 0.09 | Minor |
| 108 | Delirous | Jason Derulo | American | English | Pop funk | Fast | 128 | 0.10 | Minor |
| 109 | Demons | Imagine Dragons | American | English | Pop rock | Moderate | 89 | 0.19 | Major |
| 110 | Den of thieves | The Trews | Canadian | English | Pop rock | Moderate | 116 | -0.01 | Major |
| 111 | Despacito | Luis Fonsi, Daddy Yankee | Portorican | English | Latin pop | Moderate | 111 | -0.10 | Minor |
| 112 | Despacito | Luis Fonsi, Daddy Yankee, Justin Bieber | Portorican | Spanish | Latin pop | Moderate | 85 | -0.10 | Minor |
| 113 | Did u ever think | R Kelly | American | English | Hip hop | Moderate | 89 | -0.04 | Minor |
| 114 | Do it like me | iAmDLOW | American | English | Rap/hip hop | Moderate | 76 | -0.17 | Minor |
| 115 | Dollhouse | Melanie Martinez | American | English | Pop | Fast | 131 | 0.10 | Minor |
| 116 | Don't let me down | The Chainsmokers | American | English | Pop | Moderate | 109 | -0.08 | Minor |
| 117 | Don't let me down | The Chainsmokers | American | English | Electrohouse | Moderate | 109 | -0.10 | Minor |
| 118 | Don't mind onscreen | Kent Jones | American | English | Rap | Moderate | 106 | 0.18 | Major |
| 119 | Don't stop believing | Journey | American | English | Rock | Moderate | 118 | 0.20 | Major |
| 120 | Dora the explorer theme song | Joshua Sitron, Billy Straus | American | English | Cartoons | Moderate | 119 | 0.10 | Major |
| 121 | Dream on | Aerosmith | American | English | Rock | Moderate | 79 | -0.05 | Minor |
| 122 | Duel of the fate (Star Wars) | John Williams | American | N. A. | Instrumental | Fast | 155 | 0.02 | Major |
| 123 | Dynamite | Taio Cruz | British | English | Pop | Moderate | 83 | 0.03 | Major |
| 124 | Easy lover | Philip Bailey | American | English | Pop | Fast | 129 | -0.04 | Major |
| 125 | Electric | Shawn Desman | Canadian | English | Pop | Moderate | 114 | -0.05 | Minor |
| 126 | Empire | Rococode | Canadina | English | Alternative | Moderate | 104 | 0.08 | Major |
| 127 | Enter in these gates of old | Concordia Publishing House | American | English | Church/pop | Moderate | 73 | 0.07 | Major |
| 128 | Esquece o mundo | Yasmine Carvalho | Portuguese | Portuguese | Pop | Fast | 186 | 0.21 | Major |
| 129 | Every little step | Bobby Brown | American | English | Hip hop/dance pop | Moderate | 100 | -0.18 | Minor |
| 130 | Everybody talks | Neon Trees | American | English | Rock | Moderate | 104 | 0.11 | Major |
| 131 | Everyday | Rod Stewart | British | English | Pop | Moderate | 100 | -0.06 | Minor |
| 132 | Everything is awesome (The Lego movie) | Tegan and Sara | Canadian | English | Pop dance | Fast | 148 | 0.22 | Major |
| 133 | Eye of the tiger | Survivor | American | English | Rock | Fast | 145 | -0.10 | Minor |
| 134 | Faded | Alan Walke | English | English | Pop | Moderate | 105 | -0.13 | Minor |
| 135 | Fairly OddParents theme song | Ron Jones, Butch Hartman | American | English | Cartoons | Moderate | 104 | -0.22 | Minor |
| 136 | Fall out boy | Uma Thurman | American | English | Pop | Fast | 150 | 0.12 | Major |
| 137 | Fallen angel | Robbie Roberston | Canadian | English | Rock | Fast | 129 | -0.13 | Minor |
| 138 | Fancy | Iggy Azalean | Australian | English | Rap | Fast | 190 | -0.26 | Minor |
| 139 | Fight song | Rachel Platten | American | English | Pop | Moderate | 91 | 0.11 | Minor |
| 140 | Fireball | Pitbull | American | English | Pop | Fast | 123 | 0.01 | Minor |
| 141 | Fixing a hole | Beatles | British | English | Rock | Moderate | 106 | -0.29 | Minor |
| 142 | Flaws | Bastille | British | English | Pop | Fast | 144 | 0.06 | Major |
| 143 | Footloose | Kenny Loggins | American | English | Pop | Fast | 174 | -0.03 | Major |
| 144 | Freaks | Timmy Trumpet, Savage | Australian | English | Pop | Fast | 128 | 0.01 | Minor |
| 145 | French kiss | Black M | French | French | Rap/hip hop | Fast | 122 | 0.05 | Minor |
| 146 | Frérot | Black M, Soprano | French | French | Rap/hip hop | Fast | 135 | 0.19 | Major |
| 147 | Friends are family | Oh, Hush! | American | English | Pop rock | Moderate | 106 | 0.05 | Major |
| 148 | Friendship is magic theme song from My Little Pony | Daniel Ingram | American | English | Cartoons | Moderate | 129 | -0.22 | Minor |
| 149 | Full extreme | Ultimate Rejects | Caraibic | English | Pop | Fast | 170 | 0.02 | Minor |
| 150 | Game shaker theme song | Michael Corcoran | American | English | Pop | Fast | 141 | -0.15 | Minor |
| 151 | Gangnam style | PSY | South Corean | English | K-pop | Fast | 132 | -0.22 | Minor |
| 152 | GDFR | Flo Rida | American | English | Rap | Moderate | 97 | 0.04 | Major |
| 153 | Ginza | J Balvin | Colombian | Spanish | Pop | Moderate | 102 | -0.14 | Minor |
| 154 | Girl talking bout | Mindless Behavior | American | English | Hip hop | Fast | 123 | -0.10 | Minor |
| 155 | Girls just wanna have fun | Cyndi Lauper | American | English | Pop | Slow | 60 | -0.24 | Major |
| 156 | Girls on fire | Alicia Keys | American | English | R&B/soul | Fast | 186 | -0.28 | Minor |
| 157 | Give me love | Kyle Braun, Broderick Jones |  | English | Pop | Moderate | 100 | 0.28 | Major |
| 158 | Glad you came | The Wanted | British, Irish | English | Folk country | Moderate | 114 | -0.04 | Minor |
| 159 | Go off | Lil Uzi Vert, Quavo, Travis Scott | American | English | Rap | Moderate | 118 | -0.12 | Minor |
| 160 | Go your own way | Fleetwood Mac | British, American | English | Rock | Fast | 135 | -0.03 | Major |
| 161 | Good drank | 2 Chainz, Gucci Mane, Quavo | American | English | Rap | Fast | 130 | 0.19 | Minor |
| 162 | Good feeling | Flo Rida | American | English | Pop rap | Fast | 129 | 0.01 | Minor |
| 163 | Good to be alive | Andy Grammer | American | English | Pop | Moderate | 120 | 0.04 | Major |
| 164 | Goosebumps | Travis Scott, Kendrick Lamar | American | English | Rap | Moderate | 88 | -0.14 | Minor |
| 165 | Half way there | Bon Jovi | American | English | Rock | Fast | 121 | -0.23 | Minor |
| 166 | Hallelujah chorus from Handel's Messiah | Handel | German | English | Classic | Fast | 188 | 0.37 | Major |
| 167 | Hands to myself | Selena Gomez | American | English | Pop | Moderate | 73 | 0.18 | Major |
| 168 | Happy | Pharrell Williams | American | English | Pop | Moderate | 107 | -0.05 | Minor |
| 169 | Haunted | Bejoncè | American | English | R&B | Fast | 125 | -0.01 | Minor |
| 170 | Havana young thug | Camila Cabello | Cuban | English | Latin pop | Moderate | 105 | -0.03 | Minor |
| 171 | Head over heels | Tears for Fears | British | N. A. | New wave/pop | Moderate | 95 | 0.26 | Major |
| 172 | Heavy dirty soul | 21 Pilots | American | English | Rap | Fast | 130 | -0.23 | Minor |
| 173 | Hedwig's theme (Harry Potter and the Sorcerer's Stone) | John Williams | American | N. A. | Soundtrack | Slow | 52 | -0.32 | Minor |
| 174 | Hello | Adele | British | English | Pop | Moderate | 106 | 0.08 | Minor |
| 175 | Here comes the Sun | Beatles | British | English | Folk pop | Moderate | 88 | 0.10 | Major |
| 176 | Here I am (come and take me) | Al Green | American | N. A. | Pop/R&B | Moderate | 91 | -0.10 | Minor |
| 177 | Here I am to worship | Michael W. Smith | American | English | Church/pop | Moderate | 77 | 0.17 | Major |
| 178 | Hero | Mariah Carey, Luciano Pavarotti | American, Italian | English | Pop | Moderate | 116 | 0.05 | Minor |
| 179 | Hey there Delilah | Plain White Ts | American | English | Pop/country | Moderate | 105 | 0.24 | Major |
| 180 | Hide away | Daya | American | English | Pop | Moderate | 94 | 0.15 | Major |
| 181 | Hideaway | Kiesza | Canadian | English | Pop | Moderate | 79 | 0.13 | Major |
| 182 | Hold on (the break) | Walk Off The Earth | Canadian | English | Rock | Fast | 88 | 0.31 | Major |
| 183 | Hollaback girl | Gwen Stefani | American | English | Pop | Moderate | 110 | -0.16 | Minor |
| 184 | Honey bear | Bryant Oden | American | English | Pop | Fast | 161 | 0.16 | Major |
| 185 | Hooked on a feeling | Blue Swede | Swedish | English | Pop | Moderate | 114 | -0.03 | Minor |
| 186 | Hope | Tobu | Latvian | N. A. | Electro House | Fast | 128 | 0.19 | Major |
| 187 | Hot hot hot | Vengaboys | German | English | Pop | Moderate | 65 | 0.29 | Major |
| 188 | Hotline bling | Drake | Canadian | English | Hip hop | Moderate | 90 | -0.13 | Minor |
| 189 | How are you (greeting song) | Busy Beavers | American | English | Cartoons | Moderate | 116 | 0.07 | Major |
| 190 | How far I'll go | Alessia Cara | Canadian | English | Pop | Fast | 117 | 0.28 | Major |
| 191 | How I am supposed to live without you | Michael Bolton | American | English | Pop | Fast | 139 | 0.00 | Minor |
| 192 | How soon is now | The Smiths | British | English | Pop dance | Fast | 127 | -0.03 | Major |
| 193 | Hungry like a wolf | Duran Duran | English | English | Rock, new wave | Fast | 130 | 0.14 | Major |
| 194 | I believe I can fly | R. Kelly | American | English | Pop | Moderate | 117 | -0.01 | Major |
| 195 | I bet my life | Imagine Dragons | American | English | Pop, guitar, voice | Fast | 147 | 0.15 | Major |
| 196 | I don't wanna know | Maroon 5 | American | English | Pop | Fast | 131 | 0.11 | Major |
| 197 | I feel it coming | Canadian | Canadian | English | Pop | Moderate | 93 | -0.04 | Minor |
| 198 | I found you | The wanted | Irish | English | Dance | Fast | 129 | -0.31 | Minor |
| 199 | I gotta feeling | The Black Eyed Peas | American | English | Pop | Fast | 128 | 0.14 | Major |
| 200 | I knew you were trouble | Taylor Swift | American | English | Pop | Moderate | 103 | 0.08 | Major |
| 201 | I love it (I don't care) | Icona Pop | Swedish | English | Dance | Fast | 126 | 0.07 | Major |
| 202 | I love you song | Barney Songs | American | English | Cartoons | Fast | 173 | 0.14 | Major |
| 203 | I want it that way | Backstreet boys | American | English | Pop, dance | Moderate | 104 | 0.12 | Major |
| 204 | I want you (she's so heavy) | Beatles | British | English | Pop | Fast | 161 | -0.23 | Minor |
| 205 | I worship You, almighty God I give you my heart | Teo Poh Heng | Singaporean | English | Church/pop | Moderate | 71 | 0.15 | Major |
| 206 | I'll make some cake | ItsAllMinecraft | American | English | Pop | Fast | 124 | -0.03 | Minor |
| 207 | I'm a bun (from "The amazing world of Gumball") | Dan Russell | American | English | Cartoons | Fast | 213 | -0.05 | Major |
| 208 | I'm a teenager | N. A. | Russian | Hebrew | Pop ethnic | Fast | 142 | -0.09 | Minor |
| 209 | I'm an albatraoz | AronChupa | Swedish | English | House | Fast | 128 | -0.16 | Minor |
| 210 | I'm bringing home a baby bumblebee | Evokids | German | English | Cartoons | Fast | 166 | 0.19 | Major |
| 211 | I'm happy song | Busy Beavers | American | English | Cartoons | Fast | 122 | 0.18 | Major |
| 212 | I'm not the only one | Sam Smith | English | English | Pop | Moderate | 82 | -0.02 | Minor |
| 213 | I'm not the only one | Sam Smith | British | English | Pop | Moderate | 82 | -0.09 | Minor |
| 214 | I'm singing in the rain | Gene Kelly | American | English | Pop | Fast | 123 | 0.15 | Major |
| 215 | I'm still standing | Elton John | British | English | Pop | Fast | 175 | 0.13 | Major |
| 216 | I'm the one | DJ Khaled | American | English | Hip Hop | Slow | 65 | 0.21 | Major |
| 217 | Immortals | Fall Out Boy | American | English | Pop | Moderate | 107 | -0.15 | Minor |
| 218 | Indian summer | Jai Wolf | American | English | Pop/disco | Fast | 174 | 0.12 | Major |
| 219 | Infectious | Tobu | Latvian | N. A. | Electro House | Fast | 128 | 0.10 | Major |
| 220 | Inner ninja | Classified | Canadian | English | Hip hop | Moderate | 113 | 0.02 | Major |
| 221 | Inside the vogon ship | Disney | American | English | Soundtrack | Moderate | 116 | -0.12 | Minor |
| 222 | Invincible | Deaf Kev | German | N. A. | Instrumental | Fast | 136 | 0.21 | Major |
| 223 | Invisible (RED) | U2 | Irish | English | Rock | Fast | 137 | 0.16 | Major |
| 224 | Iron man | Black Sabbath | British | English | Punk Rock | Slow | 64 | -0.04 | Minor |
| 225 | iSpy | KYLE, Lil Yachty | American | English | Rap/hip hop | Moderate | 75 | 0.01 | Major |
| 226 | It ain't me | Kygo, Selena Gomez | Norse | English | Pop | Moderate | 98 | -0.01 | Minor |
| 227 | IT trailer music (2017 movie) | Benjamin Wallfisch | British | N. A. | Soundtrack | Fast | 128 | 0.16 | Major |
| 228 | It's all on u | Illenium, Liam O'Donnell | American | English | Pop | Moderate | 109 | -0.15 | Minor |
| 229 | I've got you under my skin | Frank Sinatra | American | English | Jazz | Fast | 124 | 0.07 | Major |
| 230 | Jesus loves me | Listener Kids | American | English | Church/pop/folk | Moderate | 119 | 0.11 | Major |
| 231 | Johny Johny (Yes papa cartoon) | Nursery Rhyme | American | English | Cartoons | Moderate | 79 | 0.19 | Major |
| 232 | Jordan Belfort | Wes Walker, Dyl | American | English | Rap | Moderate | 80 | -0.08 | Minor |
| 233 | Juju on that beat | Zay Hilfigerrr & Zayion McCall | American | English | Rap/hip hop | Moderate | 107 | -0.06 | Minor |
| 234 | Jump in the line | Harry Belafonte | American | English | Reggae | Moderate | 116 | 0.16 | Major |
| 235 | Just like fire | P!nk | American | English | Soundtrack | Moderate | 83 | 0.24 | Major |
| 236 | Just the way you are | Bruno Mars | American | English | Pop | Moderate | 109 | 0.15 | Major |
| 237 | Kids | MGMT | American | English | Rock | Fast | 123 | -0.03 | Major |
| 238 | King of wishful thinking | Go West | British | English | Alternative rock | Moderate | 108 | 0.11 | Major |
| 239 | Knock me off my feet | Dan Talevski | Canadian | English | Pop | Fast | 120 | 0.00 | Major |
| 240 | Kylo Ren (Star Wars) | John Williams | American | N. A. | Instrumental | Moderate | 114 | -0.17 | Minor |
| 241 | La Maza en vivo | Mercedes Sosa | Argentinian | Spanish | Pop | Moderate | 111 | -0.12 | Minor |
| 242 | Last day | N. A. | Russian | Hebrew | Pop ethnic | Fast | 126 | -0.07 | Minor |
| 243 | Leave me alone | Michael Jackson | American | English | Funk pop | Fast | 123 | 0.18 | Minor |
| 244 | Lego house | Ed Sheeran | British | English | Pop, voice | Fast | 158 | 0.07 | Major |
| 245 | Let it go | Idina Menzel | American | English | Pop | Moderate | 89 | 0.03 | Major |
| 246 | Let mee love you | DJ Snake ft Justin Bieber | French | English | Techno | Fast | 133 | 0.27 | Major |
| 247 | Life | Tobu | Latvian | N. A. | Electro house | Fast | 128 | 0.09 | Major |
| 248 | Life is a highway (Cars 2006 movie) | Rascal Flatts | American | English | Rock | Moderate | 103 | 0.16 | Major |
| 249 | Like a Rolling Stone | The Rolling Stones | British | English | Pop | Fast | 184 | 0.09 | Major |
| 250 | Like an enderman | ThnxCya | British | English | Pop | Fast | 132 | -0.30 | Minor |
| 251 | Like me like I am | Traditional | Hebrew | Hebrew | Voice, guitar | Moderate | 103 | -0.13 | Minor |
| 252 | Lithium | Nirvana | American | English | Rock | Moderate | 83 | -0.13 | Major |
| 253 | Little black rain cloud (Winnie the Pooh movie) | Disney | American | English | Waltz, cartoons | Moderate | 110 | 0.11 | Major |
| 254 | Living well is best revenge | REM | American | English | Rock | Fast | 181 | -0.10 | Major |
| 255 | Lollipop | Lil Wayne, Static | American | English | Rap | Moderate | 99 | -0.02 | Minor |
| 256 | Look what you made me do | Taylor Swift | American | English | Pop | Fast | 128 | -0.22 | Minor |
| 257 | Lose yourself (8 mile movie) | Eminem | American | English | Hip hop | Slow | 64 | -0.01 | Minor |
| 258 | Lost Boy | Ruth B | Canadian | English | Pop | Slow | 59 | 0.12 | Minor |
| 259 | Love 3X | ZZ Ward | American | English | Pop | Fast | 160 | -0.02 | Minor |
| 260 | Love is an open door from Frozen movie repeat | Kristen Bell, Santino Fontana | American | English | Musical | Moderate | 107 | 0.24 | Major |
| 261 | Love me harder | Ariana Grande, The Weeknd | American | English | Pop | Moderate | 99 | 0.30 | Major |
| 262 | Love me now | John Legend | American | English | Pop | Slow | 63 | 0.17 | Major |
| 263 | Love you like a love song baby | Selena | Mexican | English | Dance pop | Moderate | 117 | -0.16 | Minor |
| 264 | Love yourself | Justin Bieber | Canadian | English | Pop | Slow | 64 | -0.04 | Minor |
| 265 | Mad hatter | Melanie Martinez | American | English | Pop | Slow | 60 | -0.28 | Minor |
| 266 | Mah-nà mah-nà Muppet Show | Piero Umiliani | Italian | N. A. | Cartoons | Moderate | 106 | -0.09 | Minor |
| 267 | Main theme (Star Wars) | John Williams | American | N. A. | Instrumental | Moderate | 109 | 0.08 | Major |
| 268 | Make me move (remix) | Culture Code, Karra | British | English | Pop | Fast | 138 | 0.17 | Major |
| 269 | Make you feel my love | Adele | English | English | Pop | Moderate | 78 | 0.04 | Minor |
| 270 | Mamma Mia | Abba | Swedish | English | Pop | Fast | 136 | -0.09 | Minor |
| 271 | Mari-Mac | Great Big Sea | Canadian | English | Folk | Fast | 128 | 0.02 | Major |
| 272 | Max and Ruby theme song | Treehouse Direct | American | English | Cartoons | Fast | 156 | 0.08 | Major |
| 273 | Me too | Meghan Trainor | American | English | Pop | Fast | 124 | -0.02 | Minor |
| 274 | Me, myself & I | G-Eazy and Bebe Rexha | American | English | Rap | Moderate | 112 | 0.18 | Minor |
| 275 | Mercy | Shawn Mendes | Canadian | English | Pop | Moderate | 139 | -0.03 | Minor |
| 276 | Mermaid | Train | American | English | Alternative rock | Moderate | 104 | -0.09 | Minor |
| 277 | Mesmerize | Tobu | Latvian | N. A. | Electro house | Fast | 128 | 0.26 | Major |
| 278 | Michael Myers theme song | John Carpenter | American | N. A. | Soundtrack | Fast | 137 | -0.04 | Minor |
| 279 | Mickey mouse march | Disney | American | English | Soundtrack | Fast | 133 | 0.03 | Major |
| 280 | Mighty fortress music thanks be to God | Concordia Publishing House | American | English | Pop/children | Fast | 130 | -0.09 | Major |
| 281 | Mighty machines theme song | Ian Graham | American | English | Cartoons | Moderate | 106 | 0.21 | Major |
| 282 | Milord | Edith Piaf | French | French | Pop | Moderate | 104 | 0.27 | Major |
| 283 | Miniature fugue 1 | Alec Rowley | British | N. A. | Instrumental | Moderate | 119 | -0.10 | Minor |
| 284 | Miniature fugue 2 | Alec Rowley | British | N. A. | Instrumental | Moderate | 96 | -0.09 | Minor |
| 285 | Miniature fugue 3 | Alec Rowley | British | N. A. | Instrumental | Fast | 124 | 0.05 | Minor |
| 286 | Minions banana song | Minions official | American | N. A. | Cartoons | Fast | 171 | -0.03 | Minor |
| 287 | Miss you | 54-40 | Canadian | English | Rock | Moderate | 97 | -0.04 | Minor |
| 288 | Mmm yeah | Austin Mahone, Pitbull | American | English | Rap | Fast | 126 | 0.13 | Major |
| 289 | Moonlight sonata (first movement) | Beethoven | German | N. A. | Classic | Moderate | 82 | -0.26 | Minor |
| 290 | Mortals | Warriyo, Laura Brehm | German | English | Pop | Moderate | 120 | -0.18 | Minor |
| 291 | Mot de passe | Damien Robitaille | Canadian | French | Rock | Fast | 174 | -0.18 | Major |
| 292 | Mourir d'aimer | Isabelle Boulay | Canadian | French | Pop | Moderate | 78 | 0.12 | Minor |
| 293 | Move like a soldier | Kristina Maria | Canadian | English | Dance pop | Fast | 130 | 0.01 | Minor |
| 294 | My house | Flo Rida | American | English | Pop | Fast | 188 | 0.06 | Major |
| 295 | My lovely name* | N. A. | Russian | Russian | Dance pop | Fast | 161 | -0.13 | Minor |
| 296 | My songs know what you did | Fall out boy | American | English | Pop funk | Fast | 152 | -0.17 | Minor |
| 297 | My type | Saint Motel | American | English | Pop | Moderate | 118 | -0.24 | Minor |
| 298 | My way | Frank Sinatra | American | English | Pop | Moderate | 106 | 0.10 | Minor |
| 299 | Narwhals | Weebl | British | English | Electro dance | Fast | 151 | 0.02 | Major |
| 300 | Neighborhood #1 | Arcade Fire | Canadian | English | Rock | Moderate | 111 | 0.06 | Major |
| 301 | Never close your eyes | Adam lambert | American | English | Pop | Fast | 134 | -0.12 | Minor |
| 302 | Never gonna give you up | Rick Astley | English | English | Pop | Moderate | 113 | 0.27 | Major |
| 303 | Never has the winter | [Valentina Tolkunova](https://mail.hollandbloorview.ca/owa/redir.aspx?C=0Z1aN2BADXtzyBnjpIoPo_iKxLaC_LFcqhG0XXfWwQr-SlSTghfXCA..&URL=https%3a%2f%2flyricstranslate.com%2fen%2fvalentina-tolkunova-lyrics.html) | Russian | Russian | Pop ethnic | Fast | 179 | -0.23 | Minor |
| 304 | New theme song | Thomas and Fr | English | English | Cartoons | Fast | 181 | 0.14 | Major |
| 305 | Night like this | Shawn Desman | Canadian | English | Pop | Moderate | 120 | -0.13 | Minor |
| 306 | No | Meghan Trainor | American | English | Pop | Fast | 188 | -0.11 | Minor |
| 307 | No soy de aquí, ni soy de allá | Facundo Cabral | Argentinian | Spanish | Folk pop | Moderate | 96 | 0.02 | Minor |
| 308 | No strings | Groundbreaking | American | English | Rock | Slow | 68 | 0.27 | Major |
| 309 | No weapon | Fred Hammond | American | English | Gospel | Slow | 57 | 0.14 | Major |
| 310 | Not going home | DVBBS & CMC$, Gia Koka | Canadian | English | Electro dance | Fast | 145 | 0.02 | Major |
| 311 | Nothing without love | Nate Ruess | American | English | Pop voice | Moderate | 88 | 0.24 | Major |
| 312 | Nuclear family | Green Day | American | English | Rock | Fast | 176 | 0.05 | Major |
| 313 | Numb chucks theme song | Graeme Cornies | Canadian | English | Cartoons | Fast | 190 | -0.01 | Minor |
| 314 | Numba 1 (tide is high) | Kardinal Offishall, Keri Hilson | Canadian | English | Pop | Moderate | 107 | 0.06 | Major |
| 315 | Numbers 1, 2, 3 | The Kids Club | American | N. A. | Cartoons | Moderate | 80 | -0.03 | Minor |
| 316 | Octopus's garden | Beatles | British | English | Pop | Moderate | 89 | 0.09 | Major |
| 317 | Oh be careful little eyes what you see | Cedarmont Kids | American | English | Children | Fast | 126 | 0.00 | Major |
| 318 | Oh Hanukkah | Traditional | Hebrew | English | Pop | Fast | 176 | 0.16 | Major |
| 319 | Old MacDonald had a farm | Nursery Rhyme | American | English | Pop | Moderate | 115 | 0.10 | Major |
| 320 | On the run | Pink Floyd | British | N. A. | Rock | Moderate | 82 | -0.01 | Major |
| 321 | One dance | Drake | Canadian | English | Rap | Moderate | 104 | 0.02 | Major |
| 322 | One less lonely girl | Justin Bieber | Canadian | English | Pop | Moderate | 116 | 0.31 | Major |
| 323 | One more time | Daft Punk | French | English | House | Fast | 123 | 0.15 | Major |
| 324 | Open the eyes of my heart lord | Paul Baloche | American | English | Pop | Moderate | 95 | -0.01 | Major |
| 325 | Ophelia | The Lumineers | American | English | Folk | Moderate | 75 | -0.16 | Minor |
| 326 | Packt like sardines in a crushed tiny box | Radiohead | British | N. A. | Alternative | Slow | 62 | -0.01 | Minor |
| 327 | Panda | Desiigner | American | English | Rap | Moderate | 97 | 0.02 | Major |
| 328 | Paris | The Chainsmokers | American | English | Pop | Moderate | 100 | 0.15 | Minor |
| 329 | Part of me | Katy Perry | American | English | Pop | Fast | 130 | -0.07 | Minor |
| 330 | Party in the USA | Miley Cyrus | American | English | Electropop | Fast | 191 | 0.14 | Minor |
| 331 | Party monster | The Weeknd | Canadian | English | Pop | Moderate | 79 | 0.17 | Minor |
| 332 | Party rock anthem | LMFAO | American | English | Rock | Moderate | 110 | 0.25 | Major |
| 333 | Peanut butter jelly time | The Buckwheat Boys | American | English | Cartoons | Fast | 154 | -0.01 | Minor |
| 334 | People are people | Depeche Mode | British | English | Pop | Fast | 121 | -0.10 | Minor |
| 335 | Phonics song 2 | Kids TV 123 | American | English | Children | Fast | 130 | -0.09 | Minor |
| 336 | Piano man | Billy Joel | American | N. A. | Piano rock | Fast | 171 | 0.25 | Major |
| 337 | PIKOTARO - PPAP (Pen Pineapple Apple Pen) | Daimaō Kosaka | Japanese | English | Pop electro | Fast | 136 | -0.19 | Minor |
| 338 | Pillowtalk | Zayn Malik | English | English | R&B | Slow | 62 | -0.06 | Minor |
| 339 | Planes - nothing can stop me now | Mark Holman | American | English | Pop | Moderate | 113 | 0.13 | Major |
| 340 | Pokemon GO | Junichi Masuda | Japanese | N. A. | Soundtrack | Fast | 128 | 0.02 | Major |
| 341 | Poker face | Lady Gaga | American | English | Pop | Moderate | 119 | -0.21 | Minor |
| 342 | Political rap | Jeremy Shada | American | English | Rap | Fast | 160 | -0.18 | Minor |
| 343 | Pompei | Bastille | British | English | Indie rock | Fast | 130 | 0.28 | Major |
| 344 | Praise Him, praise Him all ye little children | Integrity Kids | British | English | Church/pop | Moderate | 72 | 0.15 | Major |
| 345 | Pre | Earl Sweatshirt | American | English | Hip hop | Fast | 198 | -0.10 | Minor |
| 346 | Qian qian | Chinese Music | Chinede | N. A. | Ethnic | Moderate | 89 | -0.01 | Minor |
| 347 | Quando m'en vo'soletta la bohème | Anna Netrebko | Russian | Italian | Classic | Moderate | 120 | 0.17 | Minor |
| 348 | Radioactive | Imagine Dragons | American | English | Alternative rock | Slow | 67 | -0.01 | Minor |
| 349 | Rainbow connection from the Muppet movie | Steve Whitmire | American | English | Soundtrack | Moderate | 116 | 0.08 | Major |
| 350 | Rainbow road theme from Mario Kart | Kenta Nagata | Japanese | N. A. | Soundtrack | Fast | 136 | 0.28 | Major |
| 351 | Ready for it | Taylor Swift | American | English | Pop - grunge | Moderate | 107 | -0.11 | Minor |
| 352 | Real gone (Cars 2006 movie) | Sheryl Crow | American | English | Rock | Moderate | 118 | -0.14 | Major |
| 353 | Red solo cup | Toby Keith | American | English | Country | Moderate | 82 | -0.05 | Minor |
| 354 | Renegades | X Ambassadors | American | English | Pop | Fast | 180 | 0.19 | Major |
| 355 | Respect each other * | N. A. | Russian | Hebrew | Pop ethnic | Fast | 130 | -0.14 | Minor |
| 356 | Revolution | Diplo | American | English | Pop | Fast | 148 | -0.16 | Minor |
| 357 | Rey's theme (Star Wars) | John Williams | American | N. A. | Instrumental | Moderate | 103 | -0.28 | Minor |
| 358 | Ride | 21 Pilots | American | English | Pop | Moderate | 75 | 0.20 | Major |
| 359 | Ring around the rosy | The Countdown Kids | Canadian | English | Cartoons | Fast | 121 | 0.27 | Major |
| 360 | Ring of Fire | Johnny Cash | American | English | Rock | Moderate | 80 | -0.08 | Minor |
| 361 | Riptide | Vance Joy | Australian | English | Folk country | Moderate | 99 | -0.02 | Minor |
| 362 | Ritual | Marshmello ft Wrabel | American | English | Electro dance | Moderate | 111 | -0.07 | Major |
| 363 | Roar | Katy Perry | American | English | Pop | Fast | 179 | 0.17 | Major |
| 364 | Rockit | Herbie Hancock | American | N. A. | Funk | Moderate | 119 | -0.27 | Minor |
| 365 | Rolex | Ayo &Teo | American | English | Rap hip hop | Moderate | 72 | -0.14 | Minor |
| 366 | Route 66 (Cars 2006 movie) | John Mayer | American | English | Rock | Fast | 167 | -0.07 | Major |
| 367 | Ruby, don't take your love to town | Kenny Rogers | American | English | Country | Moderate | 107 | 0.05 | Major |
| 368 | Rude | Magic! | Canadian | English | Reggae | Fast | 144 | 0.12 | Major |
| 369 | Runnin' (lose it all) | Naughty Boy | English | English | Pop | Slow | 71 | 0.06 | Minor |
| 370 | Same Old Love | Selena Gomez | American | English | Pop | Moderate | 99 | -0.06 | Minor |
| 371 | Sandstorm | DJ Darude | Finnish | English | Trance | Fast | 136 | -0.19 | Minor |
| 372 | Santa Claus is coming to town (Children version) | Mary Liguori | Italian | English | Pop/folk/choir | Fast | 132 | 0.07 | Major |
| 373 | Saturday - Schabat | Traditional | Hebrew | Hebrew | Ethnic | Fast | 138 | 0.20 | Minor |
| 374 | Savage mode | Metro Boomin, 21 Savage | American | English | Rap | Moderate | 93 | -0.18 | Minor |
| 375 | Sax | Fleur East | British | English | Pop | Moderate | 118 | 0.09 | Major |
| 376 | Scars to your beautiful | Alessia Cara | Canadian | English | Pop | Moderate | 91 | -0.20 | Minor |
| 377 | Science is real | They Might Be Giants | American | English | Alternative rock | Fast | 180 | -0.01 | Minor |
| 378 | Seagulls! (Stop it now) | Bad Lip Reading | American | English | Cartoons | Moderate | 120 | -0.20 | Major |
| 379 | See you again | Wiz Khalifa | American | English | Pop | Moderate | 102 | 0.20 | Minor |
| 380 | Send my love (to your new lover) | Adele | English | English | Pop | Moderate | 109 | 0.03 | Major |
| 381 | Seven eleven | Beyonce | American | English | Rap | Fast | 136 | -0.25 | Minor |
| 382 | Sever the ties | Arman Cekin, Esther Sparkes | Dutch | English | Pop | Moderate | 100 | 0.10 | Minor |
| 383 | Shake it off | Taylor Swift | American | English | Pop | Fast | 107 | -0.03 | Major |
| 384 | Shape of you | Ed Sheeran | British | English | Pop | Fast | 130 | -0.22 | Minor |
| 385 | She-Ra opening theme | Melendy Britt | American | English | Cartoons | Fast | 138 | 0.03 | Major |
| 386 | Shine | Pillar | American | English | Post Grunge | Moderate | 111 | -0.08 | Major |
| 387 | Shoo-rah | Betty Wright | American | English | R&B | Fast | 121 | 0.02 | Major |
| 388 | Shooting stars | Aero Chord, DDARK | Greek | English | Rap | Fast | 140 | -0.15 | Minor |
| 389 | Shut up and dance | Walk the Moon | American | English | Pop | Fast | 128 | 0.04 | Major |
| 390 | Side to side | Ariana Grande, Nicki Minaj | American | English | Pop | Moderate | 106 | 0.24 | Major |
| 391 | Sign of the times | Harry Styles | British | English | Pop | Slow | 60 | 0.26 | Major |
| 392 | Single ladies | Beyonce | American | English | R&B pop | Moderate | 98 | -0.10 | Minor |
| 393 | Sisters and brothers | Sidewalks prophets | American | English | Pop rock | Moderate | 103 | -0.02 | Minor |
| 394 | Sitting on the toilet | Eloina Nonnie | American | English | Voice | Fast | 155 | 0.05 | Major |
| 395 | Sleep like a baby | U2 | Irish | English | Rock | Fast | 172 | -0.02 | Minor |
| 396 | Smells like teen spirit | Nirvana | American | English | Rock | Moderate | 117 | 0.16 | Major |
| 397 | Smile | Avril Lavigne | Canadian | English | Pop punk | Fast | 140 | 0.13 | Major |
| 398 | Smoke on the water | Deep Purple | British | English | Hard Rock/metal | Moderate | 113 | -0.07 | Minor |
| 399 | So long and thanks for all the fish | Disney | American | English | Instrumental | Moderate | 110 | 0.02 | Major |
| 400 | So this is love (Cinderella movie) | Ilene Woods | American | English | classic movie | Slow | 62 | -0.01 | Minor |
| 401 | Somebody is watching me | Rockwell | American | English | Pop | Fast | 123 | 0.00 | Minor |
| 402 | Somebody like you - Golden Road | Keith Urban | Australian | English | Country pop | Moderate | 111 | 0.09 | Major |
| 403 | Somebody that I used to know | Gotye | Belgian | English | Pop rock | Slow | 64 | -0.14 | Minor |
| 404 | Someday my prince will come | Adriana Caselotti | American | English | classic movie | Slow | 65 | -0.19 | Minor |
| 405 | Something big | Shawn Mendes | Canadian | English | Pop | Moderate | 112 | -0.01 | Minor |
| 406 | Something 'bout a truck | Kip Moore | American | English | Pop country | Fast | 174 | 0.08 | Major |
| 407 | Something just like this | The Chainsmokers, Coldplay | American | English | Pop | Moderate | 107 | 0.25 | Major |
| 408 | Somewhere Over the Rainbow Scene (The Wizard of Oz 1939 movie) | Judy Garland | American | English | Pop | Slow | 68 | 0.20 | Major |
| 409 | Sonatine, Op 30 - I Allegrement- Harp | Marcel Tournier | French | N. A. | Classical | Moderate | 103 | -0.08 | Minor |
| 410 | Song 2 | Blur | British | English | Rock | Fast | 130 | 0.05 | Major |
| 411 | Sorry | Beyonce | American | English | Pop | Moderate | 87 | 0.09 | Minor |
| 412 | Sorry | Justin Bieber | Canadian | English | Pop | Fast | 134 | 0.18 | Minor |
| 413 | Sorry for party rocking | LMFAQ | American | English | Electro hop | Fast | 134 | 0.05 | Major |
| 414 | South park theme | PRIMUS | American | English | Rock | Fast | 144 | 0.13 | Major |
| 415 | Space | Disney | American | English | Soundtrack | Fast | 141 | -0.13 | Minor |
| 416 | Space Is Cool | Markiplier, The Gregory Brothers | American | English | Pop | Moderate | 105 | 0.14 | Major |
| 417 | Space oddity | David Bowie | British | English | Progressive rock | Moderate | 90 | 0.04 | Minor |
| 418 | Spirits | The Strumbellas | Canadian | English | Rock | Moderate | 81 | 0.02 | Major |
| 419 | SpongeBob SquarePants | Nickelodeon | American | English | Cartoons | Moderate | 118 | 0.15 | Major |
| 420 | Starboy | The Weeknd, Daft Punk | Canadian | English | Pop | Moderate | 108 | 0.06 | Major |
| 421 | Stitches | Shawn Mendes | Canadian | English | Pop | Fast | 149 | 0.11 | Minor |
| 422 | Stole my heart | One Direction | British | English | Dance pop | Fast | 127 | 0.15 | Major |
| 423 | Stressed out | 21 Pilots | American | English | Rap | Fast | 171 | -0.09 | Minor |
| 424 | Stronger than you | Estelle | British | English | Rap | Fast | 133 | -0.13 | Minor |
| 425 | Stuck in the middle with you | Stealers Wheel | Scottish | English | Rock | Fast | 124 | 0.10 | Major |
| 426 | Stupid hoe | Nicki Minaj | Trinidadian | English | Rap | Fast | 125 | 0.16 | Major |
| 427 | Style | Taylor Swift | American | English | Pop | Slow | 48 | -0.08 | Minor |
| 428 | Suck my kiss | Red Hot Chili Peppers | American | English | Rock | Fast | 135 | 0.00 | Major |
| 429 | Sugar | Maroon 5 | American | English | Pop | Moderate | 81 | 0.07 | Minor |
| 430 | Summer of 69 | Bryan Adams | Canadian | English | Rock | Fast | 139 | 0.02 | Major |
| 431 | Sun goes down | Fabian Mazur | Danish | English | disco | Fast | 151 | -0.11 | Minor |
| 432 | Sunburst | Itro & Tobu | Latvian |  | Electro House | Fast | 128 | 0.37 | Major |
| 433 | Super Mario soundtrack - videogame | Koji Kondo | Japanese | English | Cartoons | Fast | 133 | 0.30 | Major |
| 434 | Superbass | Nicki Minaj | American | English | R&B/Soul | Moderate | 85 | 0.03 | Major |
| 435 | Superman theme | John Williams | American |  | Contemporary classical | Fast | 121 | -0.07 | Major |
| 436 | Sweatshirt | Jacob Sartorius | American | English | Pop | Fast | 128 | 0.17 | Major |
| 437 | Sweet child of mine | Guns 'n' Roses | American | English | Rock | Fast | 127 | 0.08 | Major |
| 438 | Sweet dreams (Goodnight Song) | Super Simple Songs | Canadian | English | Cartoons | Moderate | 118 | 0.17 | Minor |
| 439 | Sweet home Alabama | Lynyrd Skynyrd | American | English | Pop | Fast | 129 | 0.25 | Major |
| 440 | Sweet victory | Van Halen | American | English | Pop | Slow | 62 | 0.05 | Major |
| 441 | Symbolism | Electro-Light | Scottish | N. A. | Electro House | Moderate | 114 | 0.10 | Major |
| 442 | Symphony no. 9 (Scherzo) | Beethoven | German | N. A. | Classical | Fast | 181 | -0.28 | Minor |
| 443 | Take a step back | Every Avenue | American | English | Alternative Rock | Moderate | 116 | -0.08 | Minor |
| 444 | Take five | The Dave Brubeck quartet | American | N. A. | Jazz | Moderate | 83 | -0.15 | Minor |
| 445 | Take me home | Cayman Cline | American | English | Rap | Moderate | 119 | 0.01 | Major |
| 446 | Take me to church | Hozier | Irish | English | Indie Rock | Fast | 129 | -0.25 | Minor |
| 447 | Tale as old as time (Beauty and the beast movie) | Disney | American | English | Pop | Fast | 157 | 0.29 | Major |
| 448 | Ten thousand ways to die | Obituary | American | English | Funk | Fast | 186 | -0.04 | Minor |
| 449 | Thank you | MKTO | American | English | Pop- rock | Fast | 126 | -0.20 | Major |
| 450 | Thank you for saving me | Martin Smith | English | English | Alternative rock | Slow | 90 | -0.15 | Minor |
| 451 | That should be me | Justin Bieber | Canadian | English | Pop, dance pop | Slow | 68 | 0.12 | Minor |
| 452 | That’s what I like | Bruno Mars | British | English | Pop | Fast | 134 | 0.19 | Minor |
| 453 | The alphabet song | ESL Kids Games | Spain | English | Cartoons | Moderate | 116 | 0.11 | Major |
| 454 | The backyardigans theme song remix | Attic Stein | American | English | Streetpunk | Fast | 192 | -0.05 | Minor |
| 455 | The boys are back | Dropkick Murphys | American | English | Streetpunk | Fast | 140 | 0.09 | Major |
| 456 | The Christmas song | Vince Guaraldi | American | English | Jazz - piano | Fast | 186 | 0.25 | Minor |
| 457 | The cold | Exitmusic | American | English | Pop | Slow | 67 | -0.26 | Minor |
| 458 | The cure | Lady Gaga | American | English | Pop | Moderate | 112 | 0.33 | Major |
| 459 | The duck song | Bryant Oden | American | English | Cartoons | Fast | 187 | -0.03 | Major |
| 460 | The duck song 3 | Bryant Oden | American | English | Cartoons | Moderate | 105 | 0.05 | Major |
| 461 | The final countdown | Europe | Swedish | N. A. | Rock | Fast | 145 | -0.14 | Minor |
| 462 | The force theme (Star Wars Episode IV) | Meco Monardo | American | N. A. | Instrumental | Moderate | 98 | -0.07 | Minor |
| 463 | The fox (what does the fox say) | Ylvis | Norwegian | English | Pop | Fast | 128 | 0.19 | Major |
| 464 | The greatest | Sia, Kendrick Lamar | Australian | English | Pop | Fast | 128 | -0.07 | Major |
| 465 | The heart wants what it wants | Selena Gomez | American | English | Pop | Moderate | 111 | -0.09 | Minor |
| 466 | The house hunting song | Pendleton Ward | American | English | Soundtrack | Fast | 126 | -0.10 | Minor |
| 467 | The imperial march (Darth Vader's theme) | John Williams | American | N. A. | Instrumental | Moderate | 105 | -0.17 | Minor |
| 468 | The Jedi steps and finale | John Williams | American | N. A. | Instrumental | Moderate | 103 | -0.25 | Minor |
| 469 | The loud house intro | Nickelodeon | American | English | Cartoons | Fast | 185 | -0.06 | Minor |
| 470 | The miracle of Joey Ramone | U2 | Irish | English | Rock | Fast | 134 | -0.15 | Major |
| 471 | The oaf | Big Wreck | American | English | Pop- rock | Fast | 135 | -0.16 | Minor |
| 472 | The piano | EY - Bass Nation | American | English | Dance | Moderate | 107 | 0.06 | Major |
| 473 | The snake | Renée Christopher | Canadian | N.A. | Classical | Moderate | 118 | -0.11 | Minor |
| 474 | The time is now (You Can't See Me) | John Cena, Tha Trademarc | American | English | rap- reggae | Moderate | 111 | -0.13 | Minor |
| 475 | The weekend whip (LEGO Ninjago theme) | The Fold | American | English | Indie rock | Fast | 163 | 0.19 | Major |
| 476 | The Wheels On The Bus - Fun Songs for Children | LooLoo Kids | American | English | Cartoons | Fast | 136 | 0.27 | Major |
| 477 | Them bones | Alice in Chains | American | English | Metal | Fast | 164 | -0.15 | Minor |
| 478 | Thinking out loud | Ed Sheeran | British | English | Pop | Moderate | 78 | 0.13 | Major |
| 479 | This house is not for sale | Bon Jovi | American | English | Hard rock | Fast | 122 | -0.04 | Major |
| 480 | This is what you came for | Calvin Harris | British | English | Electronic dance music | Fast | 166 | 0.17 | Major |
| 481 | This life | Curtis Stigers, The Forest Rangers | American | English | Pop | Fast | 161 | -0.23 | Minor |
| 482 | Thomas the tank engine theme song | Nick Jr. | British | English | Cartoons | Moderate | 106 | 0.14 | Major |
| 483 | Three pistols | The Tragically Hip | Canadian | English | Pop | Fast | 133 | -0.04 | Minor |
| 484 | Through it all | Hillsong Worship | Australian | English | Pop | Moderate | 75 | 0.15 | Major |
| 485 | Thundershuk | AC/DC | American | English | Pop, dance | Fast | 134 | 0.04 | Major |
| 486 | Tik tok | Ke$ha | American | English | Rap - Funk | Moderate | 113 | 0.03 | Major |
| 487 | Timber | Pitbull | American | English | Pop | Fast | 130 | 0.21 | Major |
| 489 | TNT | AC/DC | Australian | English | Hard rock | Fast | 125 | -0.13 | Minor |
| 490 | TNT | CaptainSparklez | American | English | Pop | Moderate | 120 | -0.05 | Major |
| 491 | Tokyo drift | Teriyaki boyz | Japanese | English | Hip hop | Fast | 129 | -0.23 | Minor |
| 492 | Tonigh you belong to me | Eddie Vedder | American | English | Pop | Moderate | 109 | 0.05 | Major |
| 493 | TOOPY and BINOO theme song 2013 | TV Channel | Canadian | English | Cartoons | Fast | 132 | -0.09 | Major |
| 494 | Trap queen | Fetty Wap | American | English | Rap | Moderate | 99 | 0.24 | Major |
| 495 | Treat you better | Shawn Mendes | Canadian | English | Pop | Moderate | 111 | -0.07 | Minor |
| 496 | Trillian and Arthus reinvented | Disney | American | English | Soundtrack | Fast | 135 | 0.13 | Major |
| 497 | Trophies | Young Money | American | English | Hip hop | Moderate | 114 | 0.06 | Major |
| 498 | Truly madly deeply | Savage Garden | Australian | English | Pop | Fast | 167 | -0.15 | Minor |
| 499 | Trumpets | Jason Derulo | American | English | R&B | Fast | 159 | 0.14 | Major |
| 500 | Tu peux partir | Daniel Bélanger | Canadian | English | Pop | Fast | 179 | 0.13 | Major |
| 501 | Tunnel vision | Kodak Black | American | French | RAP | Moderate | 114 | -0.04 | Minor |
| 502 | Turn down for what | DJ snake | French | English | Electronic | Fast | 133 | 0.19 | Major |
| 503 | Twinkle twinkle little star | Rhymes for the Nursery | English | English | Cartoons | Moderate | 88 | 0.18 | Major |
| 504 | Uma Thurman | Fall Out Boy | American | English | Pop | Fast | 150 | 0.12 | Major |
| 505 | Under pressure | Queen | British | English | Pop | Moderate | 114 | -0.07 | Major |
| 506 | Undergound girl | Blondie | American | English | New wave | Fast | 169 | 0.01 | Major |
| 507 | Undo it | Carrie Underwood | American | English | Pop | Fast | 155 | 0.04 | Major |
| 508 | Unsteady | X Ambassadors | American | English | Rock | Moderate | 116 | 0.08 | Minor |
| 509 | Uptown funk | Mark Ronson, Bruno Mars | British | English | Pop funk | Moderate | 115 | -0.08 | Minor |
| 510 | Veltvodle street music | Disney | American | English | Jazz | Fast | 176 | 0.08 | Major |
| 511 | Vertigo | U2 | Irish | English | Rock | Fast | 140 | -0.15 | Minor |
| 512 | Via dolorosa | Lea Salonga | Philippines | English | Church | Slow | 67 | -0.13 | Minor |
| 513 | Victorious | Panic! At the Disco | American | English | Pop, dance | Moderate | 110 | -0.14 | Minor |
| 514 | Victory's won85 | Concordia Publishing House | American | English | Children pop rock | Moderate | 100 | -0.08 | Major |
| 515 | Viva la vida | Coldplay | British | English | Rock | Fast | 138 | 0.34 | Major |
| 516 | Vivir mi vida | Marc Anthony | American | Spanish | Pop | Moderate | 95 | 0.34 | Major |
| 517 | Wanna be startin' somethin' | Michael Jackson | American | English | Funk pop | Fast | 163 | -0.07 | Minor |
| 518 | Wasn't expecting that | Jamie Lawson | British | English | Pop guitar | Fast | 175 | 0.16 | Major |
| 519 | Watch me (whip/nae nae) | Silentò | American | English | Rap | Fast | 140 | -0.27 | Minor |
| 520 | Waving flag | Young artists for Haiti | Canadian | English | Pop | Moderate | 68 | -0.02 | Major |
| 521 | We can't stop the beat | Miley Cirus | American | English | Pop , rock | Moderate | 80 | -0.03 | Minor |
| 522 | We don’t believe what's on TV | 21 Pilots | American | English | pop | Moderate | 120 | 0.17 | Major |
| 523 | We don't talk anymore | Charlie Puth, Selena Gomez | American | English | Pop | Moderate | 105 | 0.00 | Major |
| 524 | We exist | Arcade Fire | Canadian | English | Rock | Moderate | 116 | 0.00 | Minor |
| 525 | We will rock you | Queen | American | English | Rock | Moderate | 80 | -0.18 | Major |
| 526 | Welcome to the jungle | Guns 'n' Roses | American | N.A | Hard rock | Moderate | 111 | -0.09 | Minor |
| 527 | What a wonderful world | Louis Armstrong | American | English | Jazz | Moderate | 111 | 0.19 | Major |
| 528 | What about us | P!nk | American | English | Pop | Moderate | 114 | 0.10 | Minor |
| 529 | What do you mean | Justin Bieber | Canadian | English | Pop | Fast | 125 | 0.14 | Major |
| 530 | What is a shooting star | Louis Singer | American | English | Children pop rock | Moderate | 96 | -0.27 | Minor |
| 531 | What's up | 4 Non Blondes | American | English | Rock | Fast | 136 | 0.10 | Major |
| 532 | Where are u now | Skrillex, Diplo, Justin Bieber | American | English | Pop | Fast | 140 | 0.11 | Major |
| 533 | Where is the love | The Black Eyed Peas | American | English | Pop | Moderate | 91 | 0.23 | Major |
| 534 | Wherever I go | OneRepublic | American | English | Pop | Moderate | 97 | -0.16 | Minor |
| 535 | White iverson | Post Malone | American | English | Rap/hip hop | Moderate | 85 | 0.10 | Major |
| 536 | Who let the dogs out | Baha Men | Bahamian | English | Pop | Fast | 131 | 0.09 | Major |
| 537 | Wildest dreams | Taylor Swift | American | English | Pop | Moderate | 140 | 0.00 | Minor |
| 538 | Wings | Little mix | American | English | Pop | Moderate | 115 | 0.10 | Major |
| 539 | Winnie the Pooh theme song | Disney | American | English | Cartoons | Fast | 157 | 0.10 | Major |
| 540 | Work | Rihanna, Drake | American | English | Pop | Moderate | 92 | -0.11 | Minor |
| 541 | Work from home | Fifth Harmony | Canadian | English | pop | Moderate | 110 | 0.09 | Minor |
| 542 | Worth it | Fifth Harmony | Canadian | English | Pop/R&B | Moderate | 112 | 0.03 | Minor |
| 543 | Wrecking ball | Miley Cyrus | American | English | Pop | Fast | 121 | -0.06 | Minor |
| 544 | XO tour llif3 | Lil Uzi Vert | American | English | Rap/hip hop | Moderate | 78 | -0.34 | Minor |
| 545 | Yellow submarine | Beatles | British | English | Pop | Moderate | 110 | 0.09 | Major |
| 546 | Yesterday | Beatles | British | English | Rock | Moderate | 91 | 0.04 | Minor |
| 547 | Yo ho (a pirate's life for me) | Disney | American | N.A | Soundtrack | Fast | 121 | -0.17 | Minor |
| 548 | Yonce | Beyonce | American | English | Rap | Fast | 176 | -0.05 | Minor |
| 549 | You can't touch this | MC Hammer | American | English | Rap | Moderate | 90 | 0.09 | Major |
| 550 | You shook me all night long | AC/DC | Australian | English | Hard rock | Moderate | 118 | -0.13 | Major |
| 551 | You was right | Lil Uzi Vert | American | English | Rap/hip hop | Moderate | 82 | -0.02 | Minor |

Tempo groups: slow (40 to 72 bpm), moderate (72 to 120 bpm) and fast (120 to 208 bpm); bpm – beats per minute; Mode (v) – value given for the mode by the MIR toolbox software; N.A. - not available; EDM – Electronic Dance Music; R&B – rhythms and blues; K-pop - Korean pop; * - name of the song translated to English

*Note: Not all songs identified as familiar in visit 1 were identified as familiar in the MEG (6.7/8 in TD and 5.8/8 in ASD). As such we used the in-scanner familiarity assessment in analyses*
