# Supplementary material for: Atypical Functional Connectivity During Unfamiliar Music Listening in Children With Autism
Source: Front Neurosci. 2022 Apr 19;16:829415. doi: 10.3389/fnins.2022.829415 (PMC9063167; doi:10.3389/fnins.2022.829415)
Supplement: Supplementary file 2 [file Table_2.DOCX]

Supplementary Material

Atypical Functional Connectivity during Unfamiliar Music Listening in Children with Autism

**Carina Freitas^1,2^**^*^**, Benjamin A. E. Hunt^3,4^, Simeon Wong^3,4^, Leanne Ristic^2^, Susan Fragiadakis^2^, Stephanie Chow^2^, Alana Iaboni^2^, Jessica Brian^2,5^, Latha Soorya^6^, Joyce Chen^7^, Russell Schachar^8^, Benjamin Dunkley^3,4^, Margot J. Taylor^1,3,4,9^, Jason P. Lerch^4,10, 11^, Evdokia Anagnostou^1,2,4,5^**

*** Correspondence:** Carina Freitas: [carina.debarrosfreitas@mail.utoronto.ca](mailto:carina.debarrosfreitas@mail.utoronto.ca)

**Supplementary Table 2: List of unfamiliar songs used in this study**

| **N** | **Name of the song** | **Artist** | **Nationality** | **Language lyrics** | **Genre** | **Tempo (group)** | **Tempo** | **Mode (V)** | **Mode** |
| --- | --- | --- | --- | --- | --- | --- | --- | --- | --- |
| 1 | 1985 | Haken | English | English | Rock/metal | Moderate | 95 | 0.13 | Major |
| 2 | Dreaming Wild | Klahr & Kev | Swedish | English | Electronic | Moderate | 105 | 0.08 | Major |
| 3 | Jumbo [NCS Release] | Alex Skrindo | Danish | Instrumental | Electronic | Fast | 130 | -0.08 | Minor |
| 4 | Kick Out The Jams - | [The Portugal Japan](https://www.google.com/search?rlz=1C1GCEU_enCA821CA821&q=kick+out+the+jams+the+portugal+japan&stick=H4sIAAAAAAAAAOPgE-LRT9c3LDYwLU4ziy9R4tLP1TcwLK4oLknXUspOttLPLS3OTNYvSk3OL0rJzEuPT84pLS5JLbJKLCrJLC5ZxKqSnZmcrZBfWqJQkpGqkJWYWwxmFOQXlZSmJ-YARQoS8wD-XLqsZwAAAA&sa=X&ved=2ahUKEwj4oM6U3KPkAhVIMawKHdrNB_cQmxMoATACegQICRAL) | Japanese | English | Punk Rock | Moderate | 116 | -0.17 | Minor |
| 5 | Star Citizen Main Theme | Pedro Macedo | Portuguese | Instrumental | Orchestral | Fast | 131 | -0.08 | Minor |
| 6 | Walk on Water | Ira Losco | Maltese | English | Rap | Fast | 174 | -0.09 | Minor |
| 7 | Rock lobster | The B-52's. | American | English | New wave | Fast | 182 | -0.06 | Minor |
| 8 | 100Ms | Dave | British | English | Rap/hip hop | Moderate | 71 | -0.08 | Minor |
| 9 | 3 Wheel-ups | Kano | British | English | Rap/hip hop | Moderate | 76 | -0.12 | Minor |
| 10 | A little respect | Silence 4 | Portuguese | English | Pop | Moderate | 110 | 0.27 | Major |
| 11 | A million voices | Polina Gagarina | Russian | English | Pop | Fast | 139 | -0.05 | Minor |
| 12 | Agnus Dei | Pedro Macedo | Portuguese | Instrumental | Orchestral | Moderate | 100 | -0.27 | Minor |
| 13 | All I Have Is My Soul | Natascha St. Pier | French | English | Pop | Moderate | 95 | 0.16 | Major |
| 14 | Alphabet, Phonics Songs Beavers | Busy Beavers | American | English | Cartoons | Moderate | 111 | 0.08 | Major |
| 15 | Alquimia, segredo guardado | Carina Freitas | Portuguese | Instrumental | Classic | Fast | 153 | -0.02 | Minor |
| 16 | Amanecer | Edurne | Spanish | Spanish | Pop | Moderate | 85 | -0.19 | Minor |
| 17 | Another Life E | Afrojack, David Guetta | Dutch | English | Electronic | Fast | 145 | -0.01 | Minor |
| 18 | Atmosphere | Joy Division | English | English | Rock | Moderate | 115 | -0.07 | Major |
| 19 | Au printemps | Jacques Brel | Belgian | French | Pop | Moderate | 85 | 0.04 | Major |
| 20 | Automatic | Inverted Mountain Beats | Dutch | Instrumental | Electrohouse | Moderate | 118 | 0.25 | Major |
| 21 | Back to life | Mikkel Solnado | Portuguese | English | Pop rock | Fast | 128 | 0.14 | Major |
| 22 | Beautiful | Chip | English | English | Rap/hip hop | Moderate | 88 | -0.03 | Minor |
| 23 | Beauty Never Lies | Bojana Stamenov | Serbian | English | Pop | Moderate | 85 | -0.16 | Minor |
| 24 | Big Man +BB2:B136 | Hyperaptive | British | English | Rap | Moderate | 95 | -0.02 | Minor |
| 25 | Black Tears | Hands on approach | Portuguese | English | Pop | Fast | 135 | 0.26 | Major |
| 26 | Blink | Cascada | German | English | Dance/Techno | Fast | 128 | 0.09 | Major |
| 27 | Blood Red Sandman | [Lordi](https://www.youtube.com/channel/UCn5gK-xJtOxYHzubFazyCKQ) | Finnish | English | Hard rock | Fast | 124 | -0.01 | Major |
| 28 | Break of Day | Edurne | Spanish | English | Pop | Fast | 123 | -0.28 | Minor |
| 29 | Breathing Deeper | Shapov & MEG/NERAK | Russian | English | Electronic | Fast | 166 | 0.12 | Major |
| 30 | Bring it back | Catherine Russell | American | English | Jazz | Moderate | 74 | -0.03 | Major |
| 31 | Built To Last | HammerFall | Swedish | English | Hard rock | Moderate | 105 | 0.00 | Minor |
| 32 | Busy for me | Aurea | Portuguese | English | Jazz | Fast | 182 | 0.03 | Minor |
| 33 | Cancao de ninar | Se acalme | Brazilian | Instrumental | Cartoons | Moderate | 90 | 0.11 | Major |
| 34 | Carcassonne | George Brassens | French | French | Pop | Fast | 136 | -0.01 | Major |
| 35 | Champions | Mikkel Solnado | Portuguese | English | Pop | Moderate | 86 | -0.09 | Minor |
| 36 | Cosmic Energy - Progressive House Mix | Fluidified | Finnish | Instrumental | Electrohouse | Fast | 170 | 0.16 | Major |
| 37 | Cosmic Energy 2 - Progressive House Mix | Fluidified | Finnish | Instrumental | Electrohouse | Fast | 128 | 0.06 | Major |
| 38 | Daisy | EZ Special | Portuguese | English | pop/rock | Fast | 147 | 0.12 | Major |
| 39 | Da-ma abraco | Miguel Gameiro | Portuguese | Instrumental | Piano | Moderate | 95 | 0.22 | Major |
| 40 | Danca dos Passaros | Antonio Pinho | Portuguese | Classical music | classical | Fast | 182 | 0.24 | Major |
| 41 | Dangerous | Cascada | German | English | Dance/Techno | Fast | 145 | 0.03 | Major |
| 42 | Days of your own | Hands on approach | Portuguese | English | Pop | Fast | 122 | -0.10 | Minor |
| 43 | Do it | Fingertips | Portuguese | Instrumental | pop | Fast | 129 | 0.10 | Major |
| 44 | Dollar bill | Screaming Trees | American | English | Grunge | Moderate | 82 | 0.13 | Major |
| 45 | Driving all night | EZ Special | Portuguese | Instrumental | Pop/rock | Moderate | 114 | -0.04 | Major |
| 46 | Duerme Negrito | Mercedes Sosa | Spanish | Spanish | Pop/Folk | Moderate | 78 | -0.08 | Major |
| 47 | Earthrise | Haken | English | English | Rock/metal | Moderate | 89 | 0.08 | Major |
| 48 | Easy to See | Feminnem | Croatian | English | Pop | slow | 64 | 0.09 | Minor |
| 49 | El arriero | Atahualpa Yupanqui | Argentine | Spanish | Pop/Folk | Fast | 125 | -0.19 | Minor |
| 50 | El Run Run | Estopa | Spanish | Spanish | Pop | Moderate | 86 | 0.22 | Major |
| 51 | Euphoria | Loreen | Swedish | English | Pop | Fast | 176 | -0.17 | Minor |
| 52 | Evacuate the Dancefloor | Cascada | German | English | Dance/Techno | slow | 64 | -0.07 | Minor |
| 53 | Fairytale | Alexander Rybak | Norwegian | English | Ethnic | Moderate | 108 | -0.23 | Minor |
| 54 | Family | Chip feat Loick Essien | English | English | Rap/hip hop | Moderate | 80 | 0.23 | Major |
| 55 | Feeling myself | Chip | English | English | Rap/hip hop | Moderate | 95 | 0.06 | Major |
| 56 | Figurative Language | Pr2A | N.A. | English | Rap | Fast | 124 | -0.02 | Minor |
| 57 | First kiss | Alexander Rybak | Norwegian | English | Pop | slow | 71 | -0.26 | Minor |
| 58 | Flashbacks | Franco | Portuguese | English | Pop | Moderate | 120 | 0.24 | Major |
| 59 | Fly on the wings of love | Olsen Brothers | Dennish | English | Pop | Moderate | 104 | 0.13 | Major |
| 60 | Give Me The Night | George Benson | American | English | Funk | Moderate | 111 | 0.06 | Minor |
| 61 | Give Your Life For Rock And Roll | [Lordi](https://www.youtube.com/channel/UCn5gK-xJtOxYHzubFazyCKQ) | Finland | English | Rock/metal | Moderate | 107 | 0.04 | Major |
| 62 | Glorious | Cascada | German | English | Dance/Techno | Fast | 128 | 0.09 | Minor |
| 63 | Golden Boy | [Nadav Guedj](https://www.youtube.com/watch?v=NdxOCTezeTg) | Israelis | English | voice | Moderate | 83 | -0.17 | Minor |
| 64 | Goodbye to Yesterday | Elina Born & Stig Rasta | Estonian | English | Pop | Moderate | 94 | 0.03 | Minor |
| 65 | Gravity | Zlata Ognevich | Ukraine | English | Pop | Fast | 170 | -0.10 | Minor |
| 66 | Gulliver Little Man of the Year | Hanna-Barbera Productions | American | Instrumental | Cartoons | Moderate | 80 | -0.21 | Minor |
| 67 | Hard Rock Hallelujah | [Lordi](https://www.youtube.com/channel/UCn5gK-xJtOxYHzubFazyCKQ) | Finnish | English | Hard rock | Fast | 123 | -0.09 | Major |
| 68 | Head In The Clouds | Gazoon Cartoons | American | English | Cartoons | Fast | 121 | 0.13 | Major |
| 69 | Hear them calling | Greta Salome | Icelander | English | Voice, guitar | Moderate | 117 | -0.35 | Minor |
| 70 | Here For You | Maraaya | Slovenian | English | Pop | Moderate | 105 | 0.01 | Minor |
| 71 | Hero March | Pedro Macedo | Portuguese | Instrumental | Orchestral | Moderate | 106 | -0.11 | Minor |
| 72 | Heroes | Mans Zelmerlow | Swedish | English | Pop | Moderate | 83 | -0.22 | Minor |
| 73 | Hour of the Wolf | Elnur Huseynov | Azerbaijani | English | Pop | Moderate | 108 | 0.22 | Major |
| 74 | I Don't Remember Your Name | Friðrik Dór | Icelandic | English | Dance/Techno | Moderate | 113 | 0.11 | Major |
| 75 | I feel like John Travolta | EZ Special | Portuguese | English | pop/rock | Moderate | 92 | 0.03 | Major |
| 76 | I miss you | Franco | Portuguese | English | Pop | Moderate | 104 | 0.25 | Major |
| 77 | I really am such a fool | EZ Special | Portuguese | English | pop | Fast | 169 | -0.09 | Major |
| 78 | I Wanna | Maria N | Latvia | English | Pop/rock | Fast | 144 | -0.14 | Minor |
| 79 | Icebraker | Agnete | Norwegian | English | House music | Fast | 163 | 0.01 | Minor |
| 80 | I'd like to walk around in your mind | Vashti Bunyan | English | English | Folk | Fast | 127 | 0.22 | Major |
| 81 | Igneous | Moon Tooth | American | Instrumental | Metal | Fast | 125 | -0.09 | Major |
| 82 | I'll remember to forget | Rita Redshoes | Portuguese | English | pop | Moderate | 104 | 0.05 | Amb. |
| 83 | I'm alive | Ethaide | Albanian | English | Ethnic | Moderate | 103 | -0.04 | Minor |
| 84 | I'm fine | Chip feat. Stormzy & Shalo | English | English | Rap/hip hop | Fast | 169 | -0.02 | Minor |
| 85 | I'm on the road to happiness | Rita Redshoes | Portuguese | English | Pop | slow | 69 | -0.01 | Major |
| 86 | In Front Of Your Eyes | [Garmiani](https://www.youtube.com/channel/UCwtzJXj2PLcu-tw6GcZQG3w) | Swedish | English | Electronic | Fast | 125 | 0.00 | Minor |
| 87 | In love with U | EZ Special | Portuguese | English | pop/rock | Fast | 135 | 0.05 | Major |
| 88 | In My Place | Ana Free | Portuguese | Instrumental | Pop | Moderate | 97 | -0.20 | Minor |
| 89 | In n'Out | EZ Special | Portuguese | English | pop/rock | slow | 121 | -0.03 | Major |
| 90 | In the Air | Chipmunk feat Keri Hilson | English | English | Rap/hip hop | Moderate | 85 | -0.10 | Minor |
| 91 | In your eyes | Niamh Kavan | Irish | English | Pop | Fast | 129 | -0.09 | Minor |
| 92 | Integration I for two pianos | Pedro Macedo | Portuguese | Instrumental | Classic | Fast | 138 | 0.10 | Major |
| 93 | Jungle 81 | Rita Redshoes | Portuguese | English | Pop | slow | 63 | -0.09 | Minor |
| 94 | Kiss me, oh kiss me | David Fonseca | Portuguese | English | Pop | Moderate | 103 | -0.24 | major |
| 95 | Le Gorille | Georges Brassens | French | French | Pop | Fast | 124 | 0.00 | Major |
| 96 | Le Petit Cheval Blanc | Georges Brassens | French | French | Pop | Fast | 125 | 0.05 | Major |
| 97 | Les bonbons | Jacques Brel | Belgian | French | Pop | Moderate | 77 | -0.02 | Major |
| 98 | Les vieux | Jacques Brel | Belgian | French | Pop | Moderate | 72 | 0.26 | Major |
| 99 | Let's be in love | Hands on approach | Portuguese | English | Pop | slow | 76 | -0.03 | Major |
| 100 | Lighthouse | Nina Kraljić | Croatian | English | Rock | Moderate | 77 | 0.14 | Major |
| 101 | Live in Peace | One Love Family | Portuguese | English | Reggae | Fast | 133 | 0.01 | Major |
| 102 | Lolek und Bolek - Die Armbrust | N. A. | Polish | Instrumental | Cartoons | Fast | 133 | 0.21 | Major |
| 103 | Lordi Devil Is A Loser | [Lordi](https://www.youtube.com/channel/UCn5gK-xJtOxYHzubFazyCKQ) | Finnish | English | Heavy Rock | Moderate | 73 | -0.02 | Minor |
| 104 | Los ejes de mi carreta | Atahualpa Yupanqui | Argentine | Spanish | Pop/Folk | Moderate | 119 | -0.29 | Minor |
| 105 | Love injected | Aminata | Latvian | English | House music | Fast | 144 | 0.07 | Major |
| 106 | Love is | Katrina Elam | American | English | Country | Slow | 72 | 0.12 | Major |
| 107 | Magical | Nina | Serbian | English | Pop | Moderate | 92 | 0.18 | Major |
| 108 | Malabares | Estopa | Spanish | Spanish | Pop | Moderate | 111 | -0.19 | Minor |
| 109 | Marble Machine | Wintergatan | Swedish | Instrumental | New Age | Moderate | 98 | 0.24 | Major |
| 110 | Maya the Bee | Karel Svoboda | German | English | Cartoons | Fast | 124 | 0.30 | Major |
| 111 | Megadeth - Tornado of Souls (HD).wav' | Megadeth | American | English | Heavy Metal | Moderate | 79 | -0.25 | Major |
| 112 | Message to my girl | Split Enz | New Zealander | English | Rock | Moderate | 104 | -0.06 | Major |
| 113 | Moskau | Dschinghis Khan | German | German | Disco | Fast | 131 | 0.17 | Major |
| 114 | Move Faster | Fingertips | Portuguese | Instrumental | Pop | Fast | 127 | -0.16 | Minor |
| 115 | Music For Monetize | Joseespirit | N.A. | English | Progressive House | Fast | 128 | -0.06 | Minor |
| 116 | Musica Para Bebe Dormir | Cassio Toledo | N.A. | Instrumental | Cartoons | Moderate | 87 | 0.02 | Major |
| 117 | My number one | Helena Paparizou | Greek | English | Pop | Fast | 151 | -0.22 | Minor |
| 118 | My Own Beat | Leonar Andrade | Portuguese | English | Pop/rock | Moderate | 112 | -0.16 | Minor |
| 119 | My wonder moon | Hands on approach | Portuguese | English | Pop rock | Fast | 137 | 0.21 | Major |
| 120 | New Way to Go | Birgit | Estonian | English | Pop | Moderate | 72 | 0.15 | Major |
| 121 | No limits | Franco | Portuguese | English | Reggae | Fast | 175 | 0.20 | Major |
| 122 | No, No, Never | Texas Lightning | German | English | Pop | Moderate | 121 | 0.14 | Minor |
| 123 | Northern lights | Kate Boy | Swedish | English | Electropop | Moderate | 97 | 0.04 | Major |
| 124 | Numbers Counting Baby Songs, Nursery Rhymes | Busy Beavers | American | English | Cartoons | Moderate | 95 | 0.12 | Major |
| 125 | Oblivion | Tavram | N.A. | Instrumental | Electrohouse | Moderate | 99 | 0.09 | Major |
| 126 | Once Again | Friðrik Dór | Icelandic | English | Pop | slow | 65 | 0.16 | Minor |
| 127 | One For Me | ByeAlex | Hungarian | English | Pop | Fast | 161 | 0.07 | Major |
| 128 | Only teardrops | Emmelie de Forest | Danish | English | Pop/Ethnic | Moderate | 111 | -0.14 | Minor |
| 129 | OPA! | Giorgos Alkaios & Friends | Greek | English | Ehtnic | Fast | 121 | -0.10 | Minor |
| 130 | Paisley | The Holydrug Couple | Chilean | English | Pop | Fast | 180 | 0.06 | Major |
| 131 | Para Llenarme De Ti | Ramón | Spanish | Spanish | Latin pop | Moderate | 102 | -0.22 | Minor |
| 132 | Pass Out | Tinie Tempah | British | English | Rap/hip hop | Moderate | 99 | -0.05 | Minor |
| 133 | Pat a mat | Lubomír Beneš | Czechoslovak | Instrumental | Cartoons | Moderate | 90 | 0.17 | Major |
| 134 | Picture of my own | Fingertips | Portuguese | Instrumental | Pop | Moderate | 123 | -0.04 | Minor |
| 135 | Planquez-vous | Keny Arkana | Argentine-French | French | Rap | Fast | 132 | -0.15 | Minor |
| 136 | Prendimi | Giovanni Allevi | Italian | Instrumental | Classical | Fast | 127 | 0.33 | Major |
| 137 | Professor Balthazar - Opening | Zlatko Grgić | Croatian | Instrumental | Cartoons | Fast | 144 | 0.13 | Major |
| 138 | Psych Out! | Garage Psyché | N.A. | English | Punk Rock | Fast | 128 | 0.31 | Major |
| 139 | Real | Of Mice and Men | American | English | Rock | Moderate | 113 | 0.05 | Major |
| 140 | Reksio - dog from Poland | Zenon Kowalowski | Polish | Instrumental | Cartoons | Fast | 137 | 0.02 | Major |
| 141 | Remember Afro House Music | DJ Manja | Portuguese | English | House music | Fast | 171 | 0.20 | Major |
| 142 | Rhythm inside | Lic Nottet | Belgium | English | Pop | Fast | 178 | -0.10 | Minor |
| 143 | Rise like a Phoenix | Conchita Wurst | Austrian | English | Pop Opera | Fast | 147 | -0.04 | Minor |
| 144 | Rumba Portuguesa | Intensa Music | Portuguese | English | Dance/Electronic | Fast | 171 | 0.07 | Major |
| 145 | Run Away | Sunstroke Project & Olia Tira | Moldovan | English | Rap | Fast | 130 | -0.03 | Minor |
| 146 | Russian Electro House 2013 Mix 70 | DJ Team Steve Strife & JayJay | Russian | Instrumental | Electrohouse | Fast | 135 | -0.05 | Minor |
| 147 | Safety dance | Men Without Hats | Canadian | English | New wave | Moderate | 106 | -0.03 | Major |
| 148 | Satellite | Lena Mayer-Landrut | German | English | Pop | Fast | 167 | -0.02 | Major |
| 149 | Scenario | A Tribe Called Quest | American | English | Rap | Fast | 169 | -0.09 | Minor |
| 150 | Sky | Alan Walker Alex Skrindo | Norwegian | Instrumental | Electronic | Fast | 130 | -0.16 | Minor |
| 151 | Snow On the Sahara | Anggun | French | English | Pop | Moderate | 112 | -0.15 | Minor |
| 152 | Some People | Shapov & Beverly Pills | Russian | English | Electronic | Fast | 122 | 0.03 | Major |
| 153 | Someone that cannot love | David Fonseca | Portuguese | English | Pop | Moderate | 103 | 0.32 | minor |
| 154 | Song from a secret garden | Cascada | German | English | Dance/Techno | Fast | 128 | 0.09 | Minor |
| 155 | Song from a secret garden | Alexander Rybak | Norwegian | English | Classic | Moderate | 118 | -0.23 | Minor |
| 156 | Starfarer (Youre the one for me | Pedro Macedo | Portuguese | Instrumental | Orchestral | Moderate | 90 | -0.14 | Minor |
| 157 | Start It Over | Club Dogo ft Cris Cab | Italian | English /Italian | Rap | Slow | 79 | -0.06 | Minor |
| 158 | Still in Love With You | Electro Velvet | British | English | Dance/Techno | Moderate | 117 | -0.14 | Minor |
| 159 | Storm pill | Moon Tooth | American | English | Metal | Fast | 185 | 0.00 | Minor |
| 160 | Sunlight | Nicky Byrne | Irish | English | Pop dance | Fast | 125 | 0.10 | Major |
| 161 | Sunshine | Vaquero | Spanish | English | Pop | Fast | 125 | 0.24 | Major |
| 162 | Suus | Rona Nishliu | Albanian | English | Jazz | Fast | 174 | -0.38 | Minor |
| 163 | Swallow my pride | Green River | American | English | Grunge | Moderate | 87 | 0.09 | Minor |
| 164 | Swing, brother, swing! | Catherine Russell | American | English | Jazz | Moderate | 110 | -0.19 | Minor |
| 165 | Tainted love | Soft Cell | British | English | Electronic | Fast | 145 | -0.02 | Major |
| 166 | Take me to your heaven | Charlotte Nilsson | Swiss | English | Pop/rock | Fast | 145 | 0.11 | Major |
| 167 | Talkin 'bout money | Fred the Godson | American | English | Rap | Moderate | 85 | -0.25 | Minor |
| 168 | Teenage Life | Daz Sampson | British | English | Rap | Fast | 180 | 0.16 | Major |
| 169 | Tennessee Tuxedo Theme song | [W. Watts Biggers](https://en.wikipedia.org/wiki/W._Watts_Biggers) | American | Instrumental | Cartoons | Fast | 140 | -0.02 | Major |
| 170 | The beginning song | Rita Redshoes | Portuguese | English | Pop | Fast | 140 | -0.09 | Minor |
| 171 | The Endless Knot | Haken | English | English | Rock/metal | Moderate | 80 | -0.18 | Minor |
| 172 | The Great Grape Ape Show | Hanna-Barbera Productions | American | Instrumental | Cartoons | Fast | 121 | -0.05 | Minor |
| 173 | The Inspector Main Theme | Henry Mancini | American | Instrumental | Cartoons | Fast | 126 | 0.22 | Major |
| 174 | The Mind's Eye | Haken | English | English | Rock | Fast | 131 | 0.22 | Major |
| 175 | The Terrorist | Dj Vadim | Russian | English | Rap/hip hop | Moderate | 105 | -0.03 | Minor |
| 176 | The Voice | Eimear Quinn | Irish | English | Ethnic/ folk | Moderate | 94 | -0.11 | Minor |
| 177 | The World Is Yours | Arch Enemy | Swedish | English | Hard rock | Fast | 185 | -0.06 | Minor |
| 178 | This Child | Kings Of Spade | American | English | Rock | Moderate | 104 | 0.00 | Major |
| 179 | Tiger Boo - English Version | Jamstar Records | American | English | Cartoons | Fast | 182 | -0.18 | Minor |
| 180 | Time | Work Drugs | American | English | Electropop | Moderate | 89 | 0.07 | Major |
| 181 | Time to Shine | Melanie Reneu | Swiss | English | Pop | Fast | 144 | -0.05 | Minor |
| 182 | Tonight again | Guy Sebastian | Australian | English | Pop | Moderate | 73 | -0.15 | Minor |
| 183 | T-shirt Weather In The Manor | Kano | British | English | Rap/hip hop | Moderate | 99 | -0.02 | Minor |
| 184 | Unbroken | Maria Olafsdottir | Icelandic | English | Pop | Fast | 188 | 0.06 | Major |
| 185 | Vilas Morenas | Antonio Pinho | Portuguese | Instrumental | classical | Moderate | 109 | -0.04 | Minor |
| 186 | Wars for Nothing | Magyar Felirat | Hungarian | English | Pop | Moderate | 88 | 0.10 | Minor |
| 187 | We Are One | One Love Family | Portuguese | English | Reggae | Fast | 164 | 0.00 | Major |
| 188 | We Are Slavic | Donatan Cleo | Polish | English | Rap | Fast | 156 | -0.11 | Minor |
| 189 | We can do anything | Gabriel | Portuguese | English | Pop | Moderate | 82 | -0.25 | Minor |
| 190 | We Could Be The Same | maNga | Turkish | English | Rock | Fast | 133 | -0.13 | Minor |
| 191 | White Lies | Rita Redshoes | Portuguese | English | Pop | Moderate | 115 | 0.11 | Major |
| 192 | Witcher 3 Main Theme | Pedro Macedo | Portuguese | Instrumental | Orchestral | Moderate | 111 | -0.12 | Minor |
| 193 | Woman, snake | Rita Redshoes | Portuguese | English | pop/progressive | Moderate | 88 | -0.03 | Minor |
| 194 | Won't let you go | Franco | Portuguese | English | Pop | Moderate | 106 | 0.15 | Major |
| 195 | Yodel It | Ilinca ft Alex Florea | Romania | English | Rap | Moderate | 87 | 0.29 | Major |
| 196 | You're gone | Fingertips | Portuguese | English | Pop | slow | 62 | 0.21 | Major |

Tempo groups: slow (40 to 72 bpm), moderate (72 to 120 bpm) and fast (120 to 208 bpm); bpm – beats per minute; Mode (v) – value given for the mode by the MIR toolbox software; N.A. - not available.
