# Supplementary material for: Atypical Functional Connectivity During Unfamiliar Music Listening in Children With Autism
Source: Front Neurosci. 2022 Apr 19;16:829415. doi: 10.3389/fnins.2022.829415 (PMC9063167; doi:10.3389/fnins.2022.829415)
Supplement: Supplementary file 3 [file Table_3.DOCX]

Supplementary Material

Atypical Functional Connectivity during Unfamiliar Music Listening in Children with Autism

**Carina Freitas^1,2^**^*^**, Benjamin A. E. Hunt^3,4^, Simeon Wong^3,4^, Leanne Ristic^2^, Susan Fragiadakis^2^, Stephanie Chow^2^, Alana Iaboni^2^, Jessica Brian^2,5^, Latha Soorya^6^, Joyce Chen^7^, Russell Schachar^8^, Benjamin Dunkley^3,4^, Margot J. Taylor^1,3,4,9^, Jason P. Lerch^4,10, 11^, Evdokia Anagnostou^1,2,4,5^**

*** Correspondence:** Carina Freitas: [carina.debarrosfreitas@mail.utoronto.ca](mailto:carina.debarrosfreitas@mail.utoronto.ca)

**Supplementary Table 3: Top 8 spatial location and extent of ALE values for contrast 1 (familiar minus unfamiliar music) and AAL labels correspondence**

| Cluster # | Volume (mm3) | ALE value | MNI | | | Side | Region | BA | AAL number | AAL  Labels |
| --- | --- | --- | --- | --- | --- | --- | --- | --- | --- | --- |
|  |  |  | ***x*** | ***y*** | ***z*** |  |  |  |  |  |
| 1 | 968 | 0.017 | 2 | 10 | 54 | Left | Superior Frontal Gyrus | 6 | 3 | Frontal Superior Left |
| 2 | 576 | 0.015 | -10 | -10 | 8 | Left | Thalamus (Ventral Lateral Nucleus) |  | 77 | Thalamus Left |
| 3 | 440 | 0.015 | 0 | 0 | 64 | Left | Medial surface of Superior Frontal Gyrus | 6 | 23 | Frontal Superior Medial Left |
| 4 | 424 | 0.012 | -52 | 10 | 14 | Left | Inferior Frontal Gyrus | 44 | 11 | Frontal Inferior Opercular Left |
| 5 | 352 | 0.014 | -30 | 18 | 6 | Left | Claustrum |  | 29 | Insula Left |
| 6 | 336 | 0.012 | -52 | -42 | 24 | Left | Superior Temporal Lobe | 13 | 81 | Temporal Superior Left. |
| 7 | 312 | 0.014 | 4 | 12 | 40 | Right | Cingulate Gyrus | 32 | 34 | Cingulum Mid Right. |
| 8 | 280 | 0.013 | -20 | 8 | -12 | Left | Lentiform Nucleus. |  | 73 | Putamen Left |
|  |  |  |  |  |  |  |  |  |  |  |

Modified version of Freitas et al (2018) ALE values for Study 1. ALE values refer to the likelihood of obtaining activation evoked by listening to familiar music stimuli in a given voxel of the standard template MRI. Coordinates are in the MNI space. Cluster #: The clusters are ranked according to their size in millimeters cubed (mm3). Abbreviations: BA, Brodmann area; x, medial-lateral; y, anterior posterior; z, superior-inferior; AAL: Automated Atlas.
