# Supplementary material for: Atypical Functional Connectivity During Unfamiliar Music Listening in Children With Autism
Source: Front Neurosci. 2022 Apr 19;16:829415. doi: 10.3389/fnins.2022.829415 (PMC9063167; doi:10.3389/fnins.2022.829415)
Supplement: Supplementary file 4 [file Table_4.DOCX]

Supplementary Material

Atypical Functional Connectivity during Unfamiliar Music Listening in Children with Autism

**Carina Freitas^1,2^**^*^**, Benjamin A. E. Hunt^3,4^, Simeon Wong^3,4^, Leanne Ristic^2^, Susan Fragiadakis^2^, Stephanie Chow^2^, Alana Iaboni^2^, Jessica Brian^2,5^, Latha Soorya^6^, Joyce Chen^7^, Russell Schachar^8^, Benjamin Dunkley^3,4^, Margot J. Taylor^1,3,4,9^, Jason P. Lerch^4,10, 11^, Evdokia Anagnostou^1,2,4,5^**

*** Correspondence:** Carina Freitas: [carina.debarrosfreitas@mail.utoronto.ca](mailto:carina.debarrosfreitas@mail.utoronto.ca)

**Supplementary Table 4: Top 8 spatial location and extent of ALE values for contrast 2 (unfamiliar minus familiar music) and AAL labels correspondence**

| Cluster # | Volume (mm3) | ALE value | MNI | | | Side | Region | BA | AAL number | AAL  Labels |
| --- | --- | --- | --- | --- | --- | --- | --- | --- | --- | --- |
|  |  |  | ***x*** | ***y*** | ***z*** |  |  |  |  |  |
| 1 | 664 | 0.012 | -38 | -24 | 16 | Left | Insula | 13 | 29 | Insula Left |
| 2 | 488 | 0.008 | 6 | 30 | 36 | Right | Cingulate Gyrus | 32 | 34 | Cingulum Mid Right |
| 3 | 176 | 0.008 | 4 | 16 | 36 | Right | Cingulate Gyrus | 32 | 34 | Cingulum Mid Right |
| 4 | 160 | 0.008 | 38 | 58 | -10 | Right | Middle Frontal Gyrus | 10 | 8 | Frontal Mid Right |
| 5 | 160 | 0.008 | 8 | -72 | 30 | Right | Precuneus | 31 | 68 | Precuneus Right |
| 6 | 152 | 0.008 | -42 | -78 | -4 | Left | Inferior Occipital Gyrus | 19 | 53 | Occipital Inferior Left. |
| 7 | 152 | 0.008 | 16 | -92 | 20 | Right | Middle Occipital Gyrus | 18 | 52 | Occipital Mid Right. |
| 8 | 152 | 0.007 | 42 | -48 | 40 | Right | Inferior Parietal Lobule | 40 | 62 | Parietal Inferior Right |

Modified version of Freitas et al (2018) ALE values for Study 2. ALE values refer to the likelihood of obtaining activation evoked by listening to unfamiliar music stimuli in a given voxel of the standard template MRI. Coordinates are in the MNI space. Cluster #: The clusters are ranked according to their size in millimeters cubed (mm3). Abbreviations: BA, Brodmann area; x, medial-lateral; y, anterior posterior; z, superior-inferior. AAL: Automated Atlas.
