# Supplementary material for: Atypical Functional Connectivity During Unfamiliar Music Listening in Children With Autism
Source: Front Neurosci. 2022 Apr 19;16:829415. doi: 10.3389/fnins.2022.829415 (PMC9063167; doi:10.3389/fnins.2022.829415)
Supplement: Supplementary file 6 [file Table_6.DOCX]

Supplementary Material

Atypical Functional Connectivity during Unfamiliar Music Listening in Children with Autism

**Carina Freitas^1,2^**^*^**, Benjamin A. E. Hunt^3,4^, Simeon Wong^3,4^, Leanne Ristic^2^, Susan Fragiadakis^2^, Stephanie Chow^2^, Alana Iaboni^2^, Jessica Brian^2,5^, Latha Soorya^6^, Joyce Chen^7^, Russell Schachar^8^, Benjamin Dunkley^3,4^, Margot J. Taylor^1,3,4,9^, Jason P. Lerch^4,10, 11^, Evdokia Anagnostou^1,2,4,5^**

*** Correspondence:** Carina Freitas: [carina.debarrosfreitas@mail.utoronto.ca](mailto:carina.debarrosfreitas@mail.utoronto.ca)

**Supplementary Table 6: Summary of all within-group network contrasts, regions of interest analysis (ROI), task >rest, threshold = 3.0.**

| Frequency-band | Measure | Condition | ASD (*p_corr_*) | Controls (*p_corr_*) |
| --- | --- | --- | --- | --- |
| Theta | wPLI | Fam > Rest | 0.217 | 1 |
|  |  | Unfam > Rest | 0.185 | 0.919 |
| Alpha | wPLI | Fam > Rest | 0.242 | 0.444 |
|  |  | Unfam > Rest | 0.865 | 1 |
| Beta | wPLI | Fam > Rest | 0.936 | 0.890 |
|  |  | Unfam > Rest | 1 | 0.108 |
| Low Gamma 1 | wPLI | Fam > Rest | 0.205 | 0.531 |
|  |  | Unfam > Rest | 0.197 | 0.220 |
| Low Gamma 2 | wPLI | Fam > Rest | 0.105 | 0.083 |
|  |  | Unfam > Rest | 0.376 | 0.008* - significant |

*p < 0.025
