# Supplementary material for: Atypical Functional Connectivity During Unfamiliar Music Listening in Children With Autism
Source: Front Neurosci. 2022 Apr 19;16:829415. doi: 10.3389/fnins.2022.829415 (PMC9063167; doi:10.3389/fnins.2022.829415)
Supplement: Supplementary file 7 [file Table_7.docx]

Supplementary Material

Atypical Functional Connectivity during Unfamiliar Music Listening in Children with Autism

**Carina Freitas^1,2^**^*^**, Benjamin A. E. Hunt^3,4^, Simeon Wong^3,4^, Leanne Ristic^2^, Susan Fragiadakis^2^, Stephanie Chow^2^, Alana Iaboni^2^, Jessica Brian^2,5^, Latha Soorya^6^, Joyce Chen^7^, Russell Schachar^8^, Benjamin Dunkley^3,4^, Margot J. Taylor^1,3,4,9^, Jason P. Lerch^4,10, 11^, Evdokia Anagnostou^1,2,4,5^**

*** Correspondence:** Carina Freitas: [carina.debarrosfreitas@mail.utoronto.ca](mailto:carina.debarrosfreitas@mail.utoronto.ca)

**Supplementary Table 7. Summary of all within-group network contrasts, whole brain analysis, task >rest, threshold = 3.0.**

| Frequency-band | Measure | Condition | ASD (*p_corr_*) | Controls (*p_corr_*) |
| --- | --- | --- | --- | --- |
| Theta | aec | Fam > Rest | 1 | 1 |
|  |  | Unfam > Rest | 1 | 1 |
| Alpha | aec | Fam > Rest | 0.814 | 0.997 |
|  |  | Unfam > Rest | 0.997 | 0.995 |
| Beta | aec | Fam > Rest | 0.573 | 0.907 |
|  |  | Unfam > Rest | 0.246 | 0.568 |
| Low Gamma 1 | aec | Fam > Rest | 0.005* - significant | 0.004* - significant |
|  |  | Unfam > Rest | 0.007* - significant | 0.006* - significant |
| Low Gamma 2 | aec | Fam > Rest | 0.062 | 0.021 |
|  |  | Unfam > Rest | 0.014* - significant | 0.003* - significant |

*p < 0.025
