# Supplementary material for: Atypical Functional Connectivity During Unfamiliar Music Listening in Children With Autism
Source: Front Neurosci. 2022 Apr 19;16:829415. doi: 10.3389/fnins.2022.829415 (PMC9063167; doi:10.3389/fnins.2022.829415)
Supplement: Supplementary file 8 [file Table_8.docx]

Supplementary Material

Atypical Functional Connectivity during Unfamiliar Music Listening in Children with Autism

**Carina Freitas^1,2^**^*^**, Benjamin A. E. Hunt^3,4^, Simeon Wong^3,4^, Leanne Ristic^2^, Susan Fragiadakis^2^, Stephanie Chow^2^, Alana Iaboni^2^, Jessica Brian^2,5^, Latha Soorya^6^, Joyce Chen^7^, Russell Schachar^8^, Benjamin Dunkley^3,4^, Margot J. Taylor^1,3,4,9^, Jason P. Lerch^4,10, 11^, Evdokia Anagnostou^1,2,4,5^**

*** Correspondence:** Carina Freitas: [carina.debarrosfreitas@mail.utoronto.ca](mailto:carina.debarrosfreitas@mail.utoronto.ca)

**Supplementary Table 8. Summary of all within-group network contrasts, whole brain analysis, task >rest, threshold = 3.0.**

| Frequency-band | Measure | Condition | ASD (*p_corr_*) | Controls (*p_corr_*) |
| --- | --- | --- | --- | --- |
| Theta | wPLI | Fam > Rest | 0.104 | 0.908 |
|  |  | Unfam > Rest | 0.267 | 0.903 |
| Alpha | wPLI | Fam > Rest | 0.53 | 0.802 |
|  |  | Unfam > Rest | 0.522 | 0.983 |
| Beta | wPLI | Fam > Rest | 0.954 | 0.864 |
|  |  | Unfam > Rest | 0.999 | 0.244 |
| Low Gamma 1 | wPLI | Fam > Rest | 0.703 | 0.144 |
|  |  | Unfam > Rest | 0.191 | 0.003* - significant |
| Low Gamma 2 | wPLI | Fam > Rest | 0.152 | 0.129 |
|  |  | Unfam > Rest | 0.036 | 0.006* - significant |

*p < 0.025
