# Supplementary material for: Atypical Functional Connectivity During Unfamiliar Music Listening in Children With Autism
Source: Front Neurosci. 2022 Apr 19;16:829415. doi: 10.3389/fnins.2022.829415 (PMC9063167; doi:10.3389/fnins.2022.829415)
Supplement: Supplementary file 9 [file Table_9.docx]

Supplementary Material

Atypical Functional Connectivity during Unfamiliar Music Listening in Children with Autism

**Carina Freitas^1,2^**^*^**, Benjamin A. E. Hunt^3,4^, Simeon Wong^3,4^, Leanne Ristic^2^, Susan Fragiadakis^2^, Stephanie Chow^2^, Alana Iaboni^2^, Jessica Brian^2,5^, Latha Soorya^6^, Joyce Chen^7^, Russell Schachar^8^, Benjamin Dunkley^3,4^, Margot J. Taylor^1,3,4,9^, Jason P. Lerch^4,10, 11^, Evdokia Anagnostou^1,2,4,5^**

*** Correspondence:** Carina Freitas: [carina.debarrosfreitas@mail.utoronto.ca](mailto:carina.debarrosfreitas@mail.utoronto.ca)

**Supplementary Table 9**: **Correlation analysis within group (ASD and TD) between networks (1, 2 or 3) connectivity strength and 3SCQ and RBS measures**

| Correlations | ASD Group | TD Group |
| --- | --- | --- |
| SCQ-total and Network 1 connectivity strength | r = 0.210; p=0.419 | r = - 0.163; p=0.491 |
| SCQ-total and Network 2 connectivity strength | r = 0.394; p=0.118 | r = 0.034; p=0.888 |
| SCQ-total and Network 3 connectivity strength | r = -0.059; p=0.821 | r = 0.077; p=0.748 |
| RBS-R (IV) and Network 1 connectivity strength | r = -0.017; p=0.950 | r = -0.016; p=0.945 |
| RBS-R (IV) and Network 2 connectivity strength | r = 0.092; p=0.725 | r = -0.061; p=0.793 |
| RBS-R (IV) and Network 3 connectivity strength | r = -0.280; p=0.277 | r = 0.180; p=0.435 |

Network 1: ROI _theta; Network 2: ROI_ beta; Network 3 = WB _theta; SCQ: Social Communication Questionnaire; RBS-R (IV): Repetitive Behaviors Scale-Revised- subscale IV (Sameness).
