# Supplementary figures and images for: Atypical Functional Connectivity During Unfamiliar Music Listening in Children With Autism
Source: Front Neurosci. 2022 Apr 19;16:829415. doi: 10.3389/fnins.2022.829415 (PMC9063167; doi:10.3389/fnins.2022.829415)

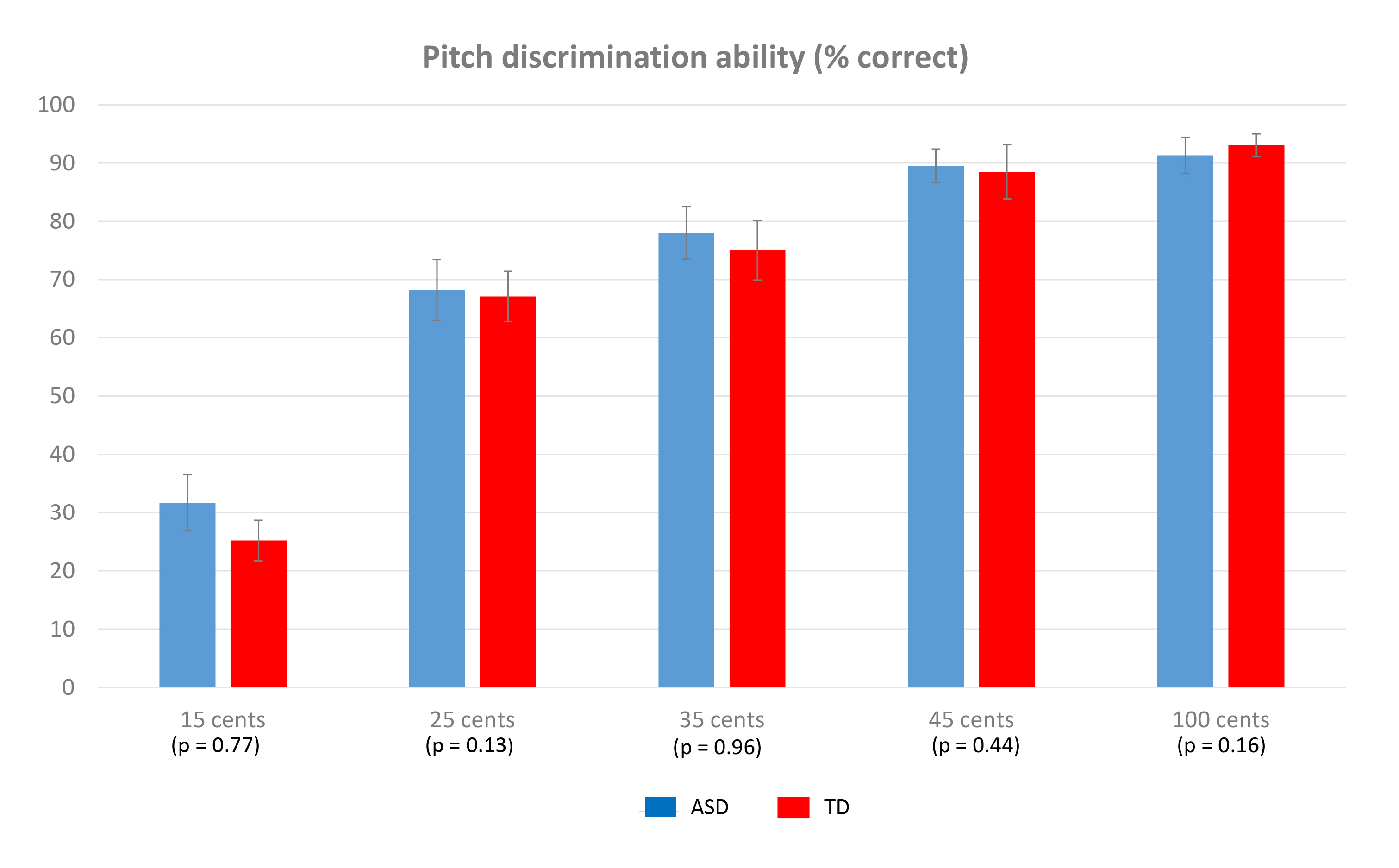

Supplement: Supplementary file 10 [file Image_1.JPEG]
